# Subchronic Cadmium-Induced Xenobiotic Toxicity in Male Wistar Rats: Antioxidant and Reproductive Protection by Standardized Silymarin with Molecular Docking Insights

**DOI:** 10.3390/jox16030103

**Published:** 2026-06-03

**Authors:** Imen Hammami, Fatma Arrari, Rahma Mahjoub, Ridha Ben Ali, Haifa El Hentati, Afef Nahdi, Eduardo Alberto López-Maldonado, Emna Talbi

**Affiliations:** 1 Laboratory of Population Health, Environmental Aggressors and Alternative Therapies (LR24ES10), Faculty of Medicine of Tunis, University of Tunis El Manar, Tunis 1007, Tunisia; 2 Department of Basic Sciences, Histology Embryology and Cell Biology Section, Faculty of Medicine of Tunis, University of Tunis El Manar, 15 Street Djebel Lakhdar, La Rabta, Tunis 1007, Tunisia; 3 Higher Institute of Biotechnology of Beja, LR: Functional Physiology and Valorization of Bio-Resources, University of Jendouba, Beja 9000, Tunisia; 4 UR17SP01-Clinical Biology Laboratory, National Institute of Nutrition and Food Technology, Tunis 1007, Tunisia; 5 National Gene Bank of Tunisia, Boulevard of the Leader Yasser Arafat, Ministry of Local Affairs and Environment, Tunis 1080, Tunisia; 6 Faculty of Chemical Sciences and Engineering, Autonomous University of Baja California, Tijuana 22424, Mexico

**Keywords:** cadmium, xenobiotics, silymarin, oxidative stress, hepatotoxicity, nephrotoxicity, reproductive toxicity, CASA, antioxidant enzymes, molecular docking

## Abstract

Cadmium is a widespread environmental xenobiotic that poses serious risks to hepatic, renal, and male reproductive functions. Natural compounds such as silymarin, a bioactive extract from *Silybum marianum*, have gained attention for their protective potential against xenobiotic-induced toxicity. This study investigated whether subchronic oral administration of silymarin (30 mg/kg) mitigates cadmium-induced toxicity (5 mg/kg) in adult rats over six weeks. Twenty-four rats were assigned to four groups: control, cadmium-exposed, silymarin-treated, and co-treated. Biochemical, hematological, oxidative stress, and reproductive parameters were assessed. Sperm quality was evaluated using CASA, and testicular tissues were examined histologically. Cadmium exposure significantly reduced body weight (−30.8%), elevated transaminases (AST, ALT; *p* < 0.01), increased serum creatinine and total cholesterol, and induced multi-organ oxidative stress, as reflected by elevated malondialdehyde and markedly reduced SOD, CAT, and thiol group levels in testicular, hepatic, and renal tissues (*p* < 0.01). Sperm concentration dropped from 75.2 to 21.8 × 10^6^/mL, with total motility falling to 35% and progressive motility to 18%, accompanied by severe seminiferous tubule degeneration (Score III in 5 rats). Co-administration of silymarin partially restored these parameters, sperm concentration recovered to 38.5 × 10^6^/mL, total motility improved to 50.2%, and antioxidant enzyme activities and liver/kidney biomarkers showed significant but incomplete recovery (*p* < 0.05). Molecular docking revealed favorable binding affinities of silybin toward GPx (−8.4 kcal/mol), CAT (−8.3 kcal/mol), and SOD (−6.4 kcal/mol), offering a preliminary computational hypothesis suggesting possible interactions between silybin and antioxidant enzymes, pending experimental validation. Silymarin alone exerted no adverse effects. These findings establish silymarin as a partial but promising multi-organ cytoprotectant against cadmium toxicity, and highlight the need for future studies optimizing dosing strategies, exploring longer treatment durations, and investigating combination approaches with metal chelators or Nrf2-activating agents to achieve complete tissue recovery.

## 1. Introduction

Potentially toxic elements (PTEs) are among the most persistent and hazardous environmental pollutants due to their toxicity, long biological half-lives, and ability to bioaccumulate in living organisms [1]. Subchronic exposure to PTEs such as arsenic, lead, mercury, and cadmium has been increasingly linked to a variety of health issues, including organ damage, immune dysfunction, endocrine disruption, and reproductive toxicity [2]. Among these, cadmium (Cd) is particularly notorious, both for its widespread environmental presence and its well-documented toxicological profile. Cadmium is a non-essential heavy metal widely used in batteries, pigments, plastic stabilizers, and electroplating industries. It can enter the human body through occupational exposure, cigarette smoke, contaminated food and water, and polluted air [3]. Once absorbed, cadmium accumulates predominantly in the liver, kidneys, and testes, where it exerts long-term toxic effects. Cadmium accumulates with a biological half-life of 10–30 years, leading to prolonged toxicity. Even at low doses (0.3–0.5 µg/kg/day), this damage is reflected by alterations in specific biomarkers, including a decreased glomerular filtration rate, increased urinary β-microglobulin excretion, and reduced fecundity, indicating early renal and reproductive dysfunction under chronic low-level exposure [4]. In biological systems, cadmium induces toxicity primarily through the generation of reactive oxygen species (ROS), which leads to oxidative stress, mitochondrial dysfunction, and lipid peroxidation. It also interferes with antioxidant defense mechanisms by depleting glutathione (GSH) and inhibiting enzymes like superoxide dismutase (SOD) and catalase (CAT). These disruptions contribute to inflammation, cell death, and tissue degeneration [5].

In the male reproductive system, cadmium impairs spermatogenesis, reduces testosterone levels, disrupts the hypothalamic–pituitary–gonadal (HPG) axis, and damages the architecture of seminiferous tubules, leading to infertility [6,7]. Similarly, in the liver and kidneys, cadmium provokes hepatocellular degeneration and nephrotoxicity, disturbing detoxification and excretory functions, as reflected by changes in transaminases and urea/creatinine levels [4,8].

Due to the absence of specific chelation therapy effective against cadmium, interest has surged in natural antioxidants capable of mitigating cadmium-induced toxicity [9]. Silymarin, a standardized extract from the medicinal plant *Silybum marianum* (milk thistle), has received attention for its potent antioxidant, anti-inflammatory, and cytoprotective effects [10]. Composed mainly of silibinin, silydianin, and silychristin, silymarin exerts protective effects via free radical scavenging, inhibition of lipid peroxidation, and upregulation of cellular antioxidant enzymes [11,12]. It also modulates inflammatory pathways by inhibiting NF-κB and MAPK activation, reducing tissue inflammation and cellular apoptosis [13]. Moreover, silymarin has shown promise in restoring hormonal balance and improving sperm parameters, including motility, count, and morphology, in models of PTEs-induced reproductive toxicity [14]. Its protective role in testicular, hepatic, and renal tissues has been validated in several preclinical models, making it a promising therapeutic candidate [15].

In the present study, the protective potential of silymarin was assessed, specifically derived from a commercially available dietary supplement, against subchronic cadmium-induced toxicity in adult male Wistar rats. The focus was placed on evaluating its effects on oxidative stress biomarkers, antioxidant enzyme activities, liver and kidney function markers, hormonal levels, and sperm quality. To complement the in vivo findings, molecular docking analyses were performed to evaluate the potential interactions of silybin, the major bioactive component of silymarin, with key antioxidant enzymes (SOD, CAT, GPx) and reproductive-related targets. This approach provides mechanistic insight into how silymarin may modulate oxidative stress pathways and contribute to the protection of testicular, hepatic, and renal tissues. Integrating docking with experimental data strengthens the biological relevance of the present study and supports the observed protective effects of the commercially available silymarin supplement. By using a marketed supplement formulation, the translational relevance of silymarin for real-world use was also explored in preventing or mitigating PTE-induced damage [16].

Despite numerous studies highlighting the protective effects of silymarin against various toxic insults, there remains a lack of data on commercially available silymarin formulations, particularly regarding their efficacy in mitigating cadmium-induced hepato-renal and reproductive toxicity. Previous research has primarily focused on pure silymarin extracts or limited exposure durations, leaving uncertainties about the optimal dose and treatment period for maximal protective effect. Therefore, this study was designed to address these gaps by evaluating a commercially derived silymarin supplement at a defined dose over a six-week period. It was hypothesized that this formulation can effectively alleviate cadmium-induced organ toxicity, and the selected dose and duration are based on prior evidence indicating sufficient bioavailability and therapeutic efficacy [17].

The cadmium dose (5 mg/kg/day) was chosen based on previous studies demonstrating its ability to induce subchronic hepato-renal and reproductive toxicity in adult male Wistar rats without causing excessive mortality [18]. The six-week exposure period was selected to allow cadmium to exert measurable organ-specific effects, consistent with subchronic toxicity models reported in the literature [19,20]. The silymarin dose (30 mg/kg/day) was based on prior evidence showing effective antioxidant and protective activity in rodents while remaining within a safe, non-toxic range; commonly used experimental doses range from 50 to 200 mg/kg/day, making 30 mg/kg/day a scientifically valid and translationally relevant regimen [21,22].

## 2. Materials and Methods

### 2.1. Preparation of Silymarin and Cadmium Chloride (CdCl_2_) Solutions

Silymarin was administered orally at a dose of 30 mg/kg body weight. The required amount was obtained from commercially available capsules (NOW^®^ brand), each containing 150 mg of standardized *Silybum marianum* extract. Each capsule was dissolved in 10 mL of distilled water to prepare a fresh stock solution daily. The administered volume was individually adjusted based on the body weight of each rat to ensure accurate dosing. The extract used in these capsules is a standardized complex of flavonolignans, primarily silybin, silychristin, and silydianin. In addition to the active compound, the formulation contains inert excipients such as cellulose (capsule), vegetable magnesium stearate, and silica, which are not known to exert pharmacological effects at the administered dose. The detailed physicochemical characteristics and composition of the formulation are provided in Table 1.

Cadmium chloride (CdCl_2_) was purchased from Sigma-Aldrich as a white crystalline powder with analytical grade purity (≥98%, molecular weight = 183.32 g/mol). The powder was stored in sealed containers, protected from moisture and light, and handled with appropriate safety precautions due to its known toxicity. A stock solution was prepared fresh daily by dissolving the appropriate amount of CdCl_2_ in distilled water to achieve a concentration allowing accurate administration of 5 mg/kg body weight per rat. The solution was prepared shortly before each administration and kept at 4 °C until use on the same day. The final volume administered was adjusted individually according to the animal’s body weight to ensure consistent dosing.

### 2.2. Animal Models

Twenty-four male albino Wistar rats, each weighing approximately 200 g, were used in the present study. At the start of the experiment, the animals were approximately 10 weeks old, corresponding to a young adult and sexually mature stage, which is appropriate for assessing reproductive and toxicological outcomes. To minimize biological variability, rats with comparable age and body weight were carefully selected and evenly distributed across the experimental groups. The animals were housed under standard laboratory conditions with a 12 h light/dark cycle, at a temperature of 22 ± 2 °C. Two rats were housed per cage, which was made of plastic with a stainless steel lid, and equipped with water bottles containing potable water. Bedding consisted of wood shavings, which were replaced every 3 to 4 days to reduce the risk of infections. Experimental groups (Cadmium, Silymarin, Cadmium + Silymarin, and Control) were identified by labeled cages. All animals were maintained in the animal facility of the Experimental Medicine Unit.

In the experimental protocol, cadmium and silymarin were administered simultaneously, once daily, via oral gavage, throughout the 6-week study period. During this period, the rats were weighed weekly. They were divided into four groups based on their treatment:

- Group C: Received by gavage of 0.9% saline solution.
- Group Sily: Received by gavage of 30 mg/kg bw of Silymarin.
- Group Cd: Received by gavage of 5 mg/kg bw of CdCl_2_.
- Group Cd + Sily: Received a co-treatment of CdCl_2_ (5 mg/kg) and Silymarin (30 mg/kg).

The oral dose of cadmium chloride (5 mg/kg bw, for six weeks) was selected based on studies showing its ability to induce marked reproductive, hepatic, and renal toxicity under subchronic exposure in rodents [23]. The silymarin dose (30 mg/kg/day) was selected based on converging evidence from the literature. Commonly used experimental doses in rodent models range from 50 to 200 mg/kg/day, making 30 mg/kg/day a conservative yet pharmacologically active regimen [21,22]. This dose has been shown to exert significant antioxidant and cytoprotective effects in models of chemically induced hepatic, renal, and reproductive toxicity without inducing adverse effects [23]. Furthermore, from a translational standpoint, this dose corresponds to a human-equivalent dose consistent with recommended therapeutic ranges for commercially available silymarin supplements, supporting the clinical relevance of the experimental design [23,24,25]. All the treatments were given in the morning (between 08:30 a.m. and 09:30 a.m.). After six weeks of treatment, rats were anesthetized with Ketamine (80 mg/kg, i.p.). Euthanasia was then performed by cervical dislocation under deep anesthesia, in accordance with ethical guidelines for laboratory animal care and the recommendations of the International Council for Laboratory Animal Sciences (ICLAS). All procedures were designed to minimize animal suffering. The 6-week duration was selected because it approximates, although does not fully encompass, the complete spermatogenic cycle in rats. In particular, when epididymal transit and sperm maturation are considered (estimated to require an additional 10–12 days), this represents an acknowledged limitation of the present study. The organs (brain, heart, liver, kidneys, testes, epididymis, prostate, and seminal vesicles) were carefully harvested and weighed. Although the primary focus of this study was on hepato-renal and reproductive toxicity, the weights of the heart and brain were also recorded as part of a general health assessment protocol routinely followed in the laboratory.

A portion of the organs was fixed in 10% formalin for histological analysis, while the remaining portion was stored at −20 °C for further studies. Blood was collected by puncture of the renal vein immediately after euthanasia of the rats, using a fine needle and sterile syringe to ensure a clean sample and avoid contamination. This method also guarantees an adequate volume of blood for hematological and biochemical analyses. The procedure was performed swiftly to minimize hemolysis and ensure reliable results.

The animal study protocol was approved by the Ethics Committee of the Faculty of Medicine of Tunis in accordance with the Standards of the International Council for Laboratory Animal Sciences (ICLAS) and local authority recommendations (protocol code CEEA-ENMV 70/23) on 26 March 2024.

### 2.3. Biochemical and Hematological Analyses

At the end of the treatment period, blood samples were collected from the renal vein immediately after euthanasia. Two types of collection tubes were used, depending on the type of analysis: For hematological assessments, blood was collected into EDTA-containing tubes (purple cap) to prevent coagulation. This allowed for a complete blood count (CBC), including hemoglobin levels, red blood cell count, total and differential white blood cell count (lymphocytes, neutrophils, eosinophils, basophils, and monocytes) [26]. For biochemical analyses, a second blood sample was collected into plain tubes without anticoagulant (red cap). After clotting, the samples were centrifuged, and the resulting serum was used for biochemical assays. The following biochemical markers were analyzed to assess organ function: creatinine, total cholesterol, HDL-cholesterol, total bilirubin, alanine aminotransferase (ALT), and aspartate aminotransferase (AST). All biochemical analyses were performed using a Beckman automated clinical system (Unicel DXC800 Synchron Clinical System, Beckman Coulter, Brea, CA, USA) at the National Institute of Nutrition and Food Technology (Tunis, Tunisia). All samples were processed within one hour of collection to ensure the accuracy and reliability of the results [27].

### 2.4. Evaluation of Sperm Parameters Using Computer-Assisted Sperm Analysis (CASA)

The evaluation of sperm parameters was performed using a Computer-Assisted Sperm Analysis (CASA) system, with the Sperm Class Analyzer^®^ (SCA^®^) software, version 5.4 (Microptic S.L., Barcelona, Spain). This system complies with the recommendations of the World Health Organization (WHO) and is adapted for sperm analysis in animal models [28]. Sperm was collected from the caudal portion of the epididymis of male rats immediately after euthanasia, performed according to ethical standards. Each caudal epididymis was excised, quickly transferred to a tube containing 1 mL of pre-warmed physiological medium at 37 °C (0.9% NaCl), and incised using a fine blade to allow sperm release. The tube was then incubated for 10 min at 37 °C to promote the spontaneous migration of sperm into the medium. After incubation, the sample was gently agitated for homogenization and then loaded into a pre-warmed standard counting chamber (Makler^®^) at 37 °C. CASA was immediately performed to prevent any alteration of motility. The SCA^®^ system automatically measured the following parameters:

1. Sperm concentration (10^6^/mL): the total number of sperm per milliliter of suspension.
2. Total motility (%): the percentage of motile sperm (progressive and non-progressive).
3. Progressive motility (%): the percentage of sperm moving in a straight line or in large arcs.
4. VAP (µm/s): Average Path Velocity, representing the speed along an average trajectory.
5. VSL (µm/s): Straight Line Velocity, measured directly from the starting point to the endpoint.
6. VCL (µm/s): Curvilinear Velocity, the actual speed following the sperm curvilinear path.

### 2.5. Oxidative Status Analysis in Hepatic, Testicular, and Renal Tissues

Each tissue (Liver, Testis, and Kidney) was homogenized in 2 mL of phosphate-buffered saline (pH 7.2) and then centrifuged at 4600 rpm for 10 min. The supernatant was collected for the analysis of oxidative stress biomarkers.

#### 2.5.1. Lipid Peroxidation (LPO) Measurement

LPO was quantified by measuring MDA using the double heating method [29]. In brief, 0.2 g of tissue homogenate was mixed with a BHT-TCA solution (1% BHT in 20% TCA), followed by centrifugation at 1000× *g* for 5 min at 4 °C. The supernatant was combined with a mixture of 0.5 N HCl and 120 mM TBA in 26 mM Tris and heated at 80 °C for 10 min. After cooling, the absorbance of the resulting chromophore was measured at 532 nm using a UV-vis spectrophotometer (Labomed, UV-2650, Fremont, CA, USA). MDA levels were calculated based on the extinction coefficient of the MDA-TBA complex (1.56 × 10^5^ M^−1^ cm^−1^), and lipid peroxidation was expressed as nmol of MDA per mg of protein.

#### 2.5.2. Thiol Group Measurement

The total concentration of thiol groups (−SH) was measured using the method of Borges and Sherma [30]. Briefly, tissue aliquots were mixed with 100 μL of 10% SDS and 800 μL of 10 mM phosphate buffer (pH 8). The absorbance was recorded at 412 nm (A0). Then, 100 μL of DTNB was added, and the mixture was incubated at 37 °C for 60 min. After incubation, the absorbance was measured again at 412 nm (A1). The thiol group concentration was calculated by subtracting A0 from A1 and using the molar extinction coefficient of 13.6 × 10^3^ M^−1^ cm^−1^. Results were expressed as nmol of thiol groups per mg of protein.

#### 2.5.3. Determination of Antioxidant Enzyme Activities in Tissue Homogenates

Superoxide dismutase (SOD) activity was assessed using a modified epinephrine assay [31]. At an alkaline pH, the superoxide anion (O_2_^−^) promotes the autoxidation of epinephrine to adenochrome. SOD inhibits this reaction, thus reducing adenochrome formation. One SOD unit is defined as the amount of enzyme that decreases adenochrome formation by 50%. The enzyme extract was mixed with a 2 mL reaction solution containing 10 μL of bovine catalase (0.4 U/μL), 20 μL of epinephrine (5 mg/mL), and 62.5 mM sodium carbonate/bicarbonate buffer (pH 10.2). Absorbance changes were measured at 480 nm. SOD activity was calculated as units per milligram of protein.

Catalase (CAT) activity was measured by monitoring the initial rate of H_2_O_2_ degradation at 240 nm [32]. The reaction mixture consisted of 30 mM H_2_O_2_ in 50 mM phosphate buffer (pH 7.0). CAT activity was calculated using the extinction coefficient of 40 mM^−1^ cm^−1^ for H_2_O_2_ and expressed as nmol of H_2_O_2_ consumed per minute per milligram of protein.

#### 2.5.4. Protein Determination

Protein concentration was measured using the Simonian and Smith assay [33], with serum albumin serving as the standard.

### 2.6. Histological Analysis

Histological processing was performed according to the method described by Fallatah et al. [34]. The aim was to obtain thin, transparent sections of testicular tissue suitable for microscopic observation following appropriate staining. To preserve the morphology as close as possible to the living state, the testes, the liver, and the kidney were fixed in 10% neutral buffered formalin for 48 h. Subsequently, the samples were dehydrated, embedded in paraffin wax, and sectioned at a thickness of 5 µm using a microtome. The tissue sections were then mounted on glass slides and stained with hematoxylin and eosin (H&E) for histological evaluation. For the testis, at least 100 seminiferous tubules per rat were analyzed under a light microscope, and the different histological injuries were classified according to the scoring method of Chen [35].

### 2.7. Molecular Docking Study

Molecular docking analysis was employed to investigate the interaction mechanisms between Silybin and selected protein targets, with particular emphasis on the types of intermolecular interactions and corresponding binding affinities. Silybin is the principal active flavonolignan component of silymarin, a standardized extract derived from the seeds of the milk thistle plant (*Silybum marianum*) [36]. To assess its antioxidant potential, three crystallographic protein structures were retrieved from the RCSB Protein Data Bank (https://www.rcsb.org; accessed on 21 May 2025): superoxide dismutase (SOD) (PDB ID: 1MFM), Catalase (CAT) (PDB ID: 1TGU) and Glutathione peroxidase (GPx) (PDB ID: 3KIJ). The 3D structure of Silybin was obtained from the PubChem database (https://pubchem.ncbi.nlm.nih.gov; accessed on 20 May 2025). All protein structures were pre-processed using AutoDock 4.2, which included the removal of co-crystallized ligands and water molecules, and the addition of Kollman partial atomic charges [37]. Molecular docking simulations were conducted within a cubic grid box of dimensions 60 × 60 × 60 Å, with a grid spacing of 0.375 Å, centered on the active sites of each target protein. The grid center coordinates (x, y, z) were specified as follows: SOD: (−13.50, 6.361, 0.833), CAT: (5.55, 2.97, 8.11), and GPx: (2.58, −8.11, 8.30). Following docking, the top-ranked ligand receptor complexes, based on binding energy and interaction stability, were further analyzed to visualize both 2D and 3D interaction patterns using Discovery Studio Visualizer 2021 [38].

### 2.8. Statistical Analysis

Statistical analysis was performed using Pythagore BIOSTAT software (version 2.0). Data were expressed as mean ± standard deviation (SD). The normality of data distribution was assessed using the Shapiro–Wilk test. Since the data did not follow a normal distribution, non-parametric tests were applied. The Kruskal–Wallis test was used to compare differences among all experimental groups, followed by the Mann–Whitney U test for pairwise comparisons. Statistical significance was set at *p* < 0.05 (*n* = 6 per group).

## 3. Results

### 3.1. Effect of Subchronic Cadmium and Silymarin Exposure on Body Weight (bw)

The evolution of body weight in rats is summarized in Table 2. Animals in the control group (C) and the group treated with Silymarin alone (Sily) exhibited a significant increase in body weight, reaching 36.8% and 43.6%, respectively, compared to their initial weights. In contrast, rats exposed to cadmium (Cd) showed a marked decrease in body weight (30.8%), and this effect was even more pronounced in the group treated with both cadmium and Silymarin (Cd + Sily), which displayed a reduction of 32.3%. These decreases reflect a loss of approximately 100 g relative to their initial weight. Statistical analysis confirmed that both the Cd and Cd + Sily groups experienced a significant reduction in final body weight compared to controls (*p* = 0.03 and *p* = 0.02, respectively). These findings highlight the toxic impact of cadmium on growth and suggest that co-administration of Silymarin does not prevent cadmium-induced weight loss.

### 3.2. Effect of Subchronic Cadmium and Silymarin Exposure on Relative Organ Weights

Table 3 summarizes the relative organ weights (g/100 g body weight) in rats. Cadmium administration significantly reduced the weights of reproductive organs, including the testes (64.3%, *p* = 0.002), epididymis (29.0%, *p* = 0.004), prostate (41.9%, *p* = 0.001), and seminal vesicles (33.8%, *p* = 0.004), when compared to controls, reflecting marked reproductive toxicity. Additionally, liver and kidney weights were reduced by 28.6% (*p* = 0.03) and 34.2% (*p* = 0.04), respectively, suggesting systemic and hepatic impairments. Heart and brain weights also showed non-significant decreases.

Rats treated with Silymarin (Sily) showed no significant changes in organ weights compared to controls. Co-administration of Silymarin with cadmium partially reversed the cadmium-induced organ weight loss. Testis, epididymis, prostate, and seminal vesicle weights increased by 26.7%, 13.6%, 11.1%, and 11.12%, respectively, compared to the Cd group. Liver and kidney weights also improved (4.0% and 20.0% increases, respectively), and slight improvements were observed in heart and brain weights.

### 3.3. Effects of Cadmium and Silymarin on Hematological and Immunological Parameters

The results of Table 4 demonstrate that exposure to cadmium (Cd) significantly impairs hematological and immunological parameters in male rats. Notably, Cd administration led to a marked decrease in lymphocyte (2.06 ± 0.05 × 10^3^/µL), monocyte (0.19 ± 0.002 × 10^3^/µL), eosinophil (0.22 ± 0.004 × 10^3^/µL), and neutrophil (0.98 ± 0.016 × 10^3^/µL) counts compared to the control group (*p* < 0.05). The co-administration of silymarin in the Cd + Sily group partially attenuated the cadmium-induced hematological alterations. Notably, the levels of lymphocytes, monocytes, eosinophils, and neutrophils were significantly increased in the Cd + Sily group compared to the Cd group, yet remained lower than those observed in the control group. These findings suggest a protective, yet incomplete, immunomodulatory effect of Silymarin against cadmium-induced leukocyte dysregulation.

Basophil counts remained relatively stable across all groups, suggesting a lesser sensitivity of this cell population to cadmium toxicity or a limited role in the response observed. Interestingly, hemoglobin concentrations were increased in the Cd and Cd + Sily groups (13.96 ± 1.91 g/dL and 14.56 ± 0.69 g/dL, respectively) compared to controls (*p* < 0.05), which may reflect a compensatory erythropoietic response or alterations in red blood cell turnover under stress conditions (Table 4).

Total immunoglobulin (Ig) levels exhibited a non-significant decrease in the Cd + Sily group. These findings suggest that, although cadmium exposure markedly alters cellular immune parameters, its effect on humoral immunity appears to be comparatively limited within the duration of exposure evaluated. Overall, these data support the hematotoxic and immunosuppressive effects of cadmium and demonstrate the ability of silymarin to confer partial protection, reinforcing its therapeutic potential against heavy metal-induced toxicity.

### 3.4. Effects of Cadmium and Silymarin on Liver Biomarkers

**Figure 1:**
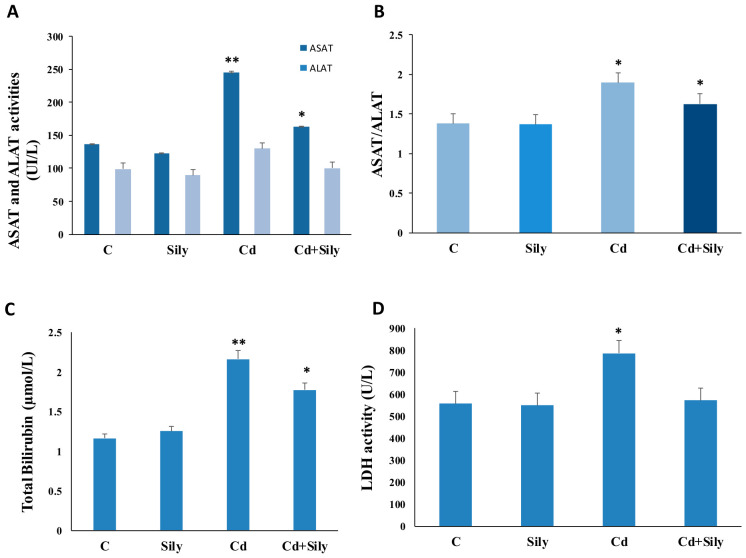
Effect of chronic treatment with Silymarin (30 mg/kg) and Cadmium (5 mg/kg) on Liver biomarkers in rats. (**A**) ASAT and ALAT activities, (**B**) ASAT/ALAT ratio, (**C**) Total Bilirubin and (**D**) LDH activity. The results are presented as the mean ± SD (*n* = 6). The stars (*) and (**) indicate significant differences compared with the control (C) group at *p* < 0.05 and *p* < 0.01, respectively.

Cadmium exposure significantly altered key liver biomarkers, indicating hepatotoxicity (Figure 1). AST and ALT activities increased markedly in the Cd group (*p* = 0.003 for AST and *p* = 0.02 for ALT), reflecting hepatocellular damage (Figure 1A). The AST/ALT ratio was also significantly elevated (*p* = 0.03), suggesting liver dysfunction with a predominant rise in AST. Silymarin treatment (Cd + Sily) partially mitigated these effects by reducing transaminase levels and slightly lowering the AST/ALT ratio, indicating a protective role (Figure 1B). Additionally, cadmium exposure led to a significant increase in total bilirubin (*p* = 0.002), highlighting impaired liver excretory function and possible hemolysis. Silymarin administration significantly decreased bilirubin levels (*p* = 0.04), suggesting an improvement in liver detoxification capacity (Figure 1C). LDH activity, a marker of tissue damage, was also significantly elevated in the Cd group (*p* = 0.03), confirming hepatocellular injury. However, silymarin treatment attenuated this increase, further supporting its hepatoprotective effect (Figure 1D).

### 3.5. Assessment of Renal Function

**Figure 2:**
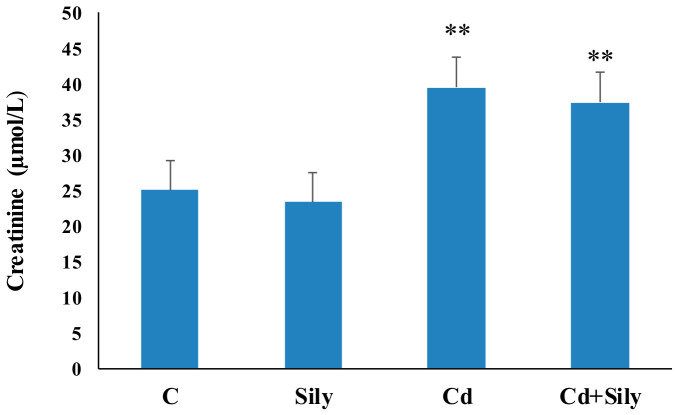
Effect of chronic oral administration of Silymarin (30 mg/kg bw) and Cadmium chloride (5 mg/kg bw) on serum creatinine levels in male rats. Data are presented as mean ± SD (*n* = 6). The results are presented as the mean ± SD (*n* = 6). The stars (**) indicate significant differences compared with the control (C) group at *p* < 0.01.

Serum creatinine levels were significantly increased in both the Cd and Cd + Sily groups compared to controls (*p* = 0.001 and *p* = 0.003, respectively), indicating impaired renal function following cadmium exposure. No significant change was observed in the Sily group, suggesting that Silymarin alone had no nephrotoxic effect (Figure 2).

### 3.6. Effect of Silymarin on Lipid Profile in Cadmium-Exposed Rats

Exposure to Cadmium (Cd) significantly altered the lipid profile of rats, increasing total cholesterol (TC), low-density lipoprotein cholesterol (LDL-C), and triglycerides (TG), while decreasing high-density lipoprotein cholesterol (HDL-C) compared to the control group. Supplementation with Silymarin (Sily) in the Cd + Sily group reduced TC, LDL-C, and TG levels, although these remained higher than in the control group. HDL-C levels showed an improvement but did not return to control levels. These results suggest that Silymarin partially mitigates lipid alterations caused by Cadmium exposure, with significant changes observed, although not fully restoring lipid profile to control levels (Table 5).

### 3.7. CASA of Sperm Parameters

**Figure 3:**
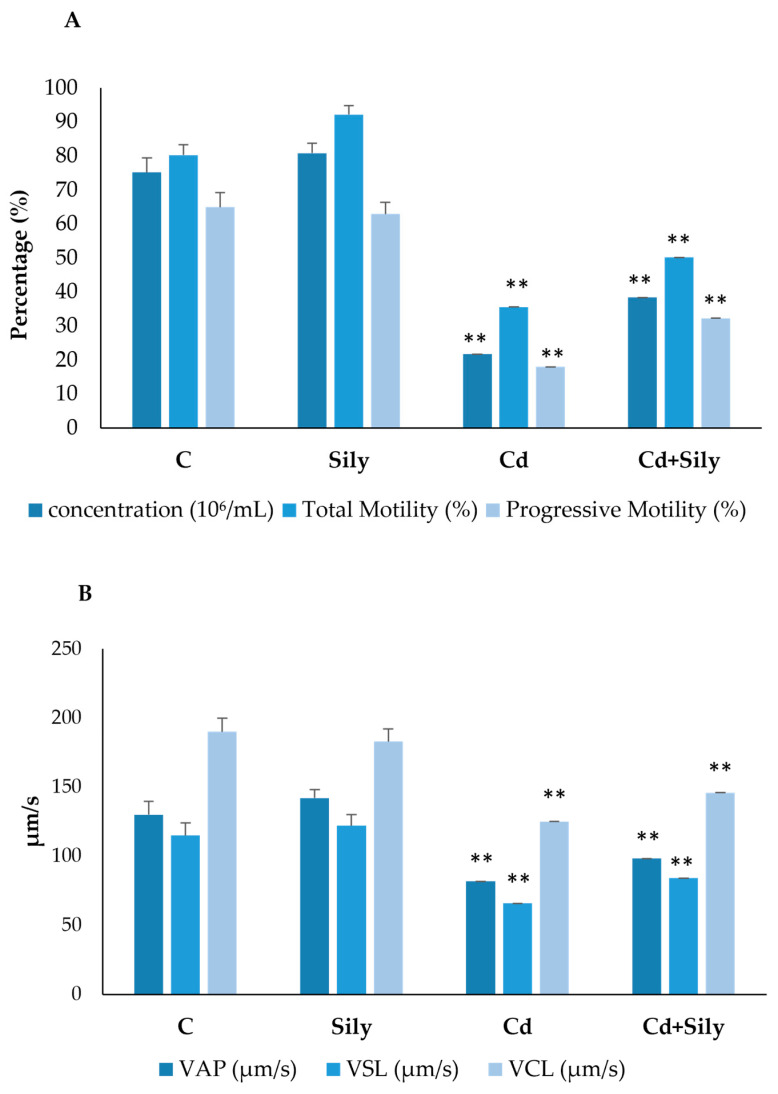
(**A**) Sperm concentration and motility parameters; (**B**) Sperm velocity parameters (VCL, VSL, VAP) evaluated using Computer-Assisted Sperm Analysis (CASA). Results are expressed as mean ± SD. C: control; Sily: silymarin; Cd: cadmium; Cd + Sily: cadmium + silymarin rats groups. The stars (**) indicate significant differences compared with the control (C) group at *p* < 0.01.

CASA revealed a significant alteration in sperm parameters in rats exposed to cadmium alone (Figure 3, Appendix A). Sperm concentration dropped to 21.80 ± 3.2 million/mL in the Cd group, compared to 75.21 ± 4.3 million/mL in the control group (*p* = 0.001). Similarly, both total and progressive motility were drastically reduced (35%, *p* = 0.004, and 18%, *p* = 0.002, respectively), accompanied by a marked decrease in VAP, VSL, and VCL velocities. The combined cadmium-silymarin (Cd + Sily) treatment showed a partial improvement in these parameters (concentration: 38.45 ± 3.91 million/mL, *p* = 0.002; total motility: 50.24%, *p* = 0.002; progressive motility: 32.33%, *p* = 0.003).

### 3.8. Effects of Cadmium and Silymarin on Oxidative Stress Biomarkers in Testis, Liver, and Kidney

**Figure 4:**
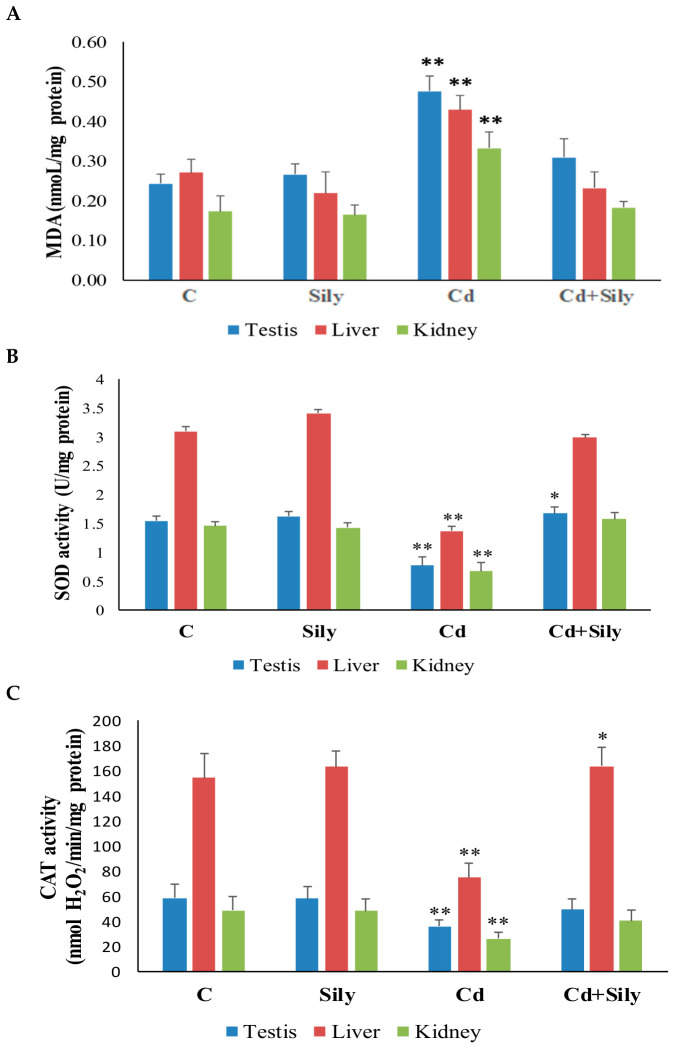

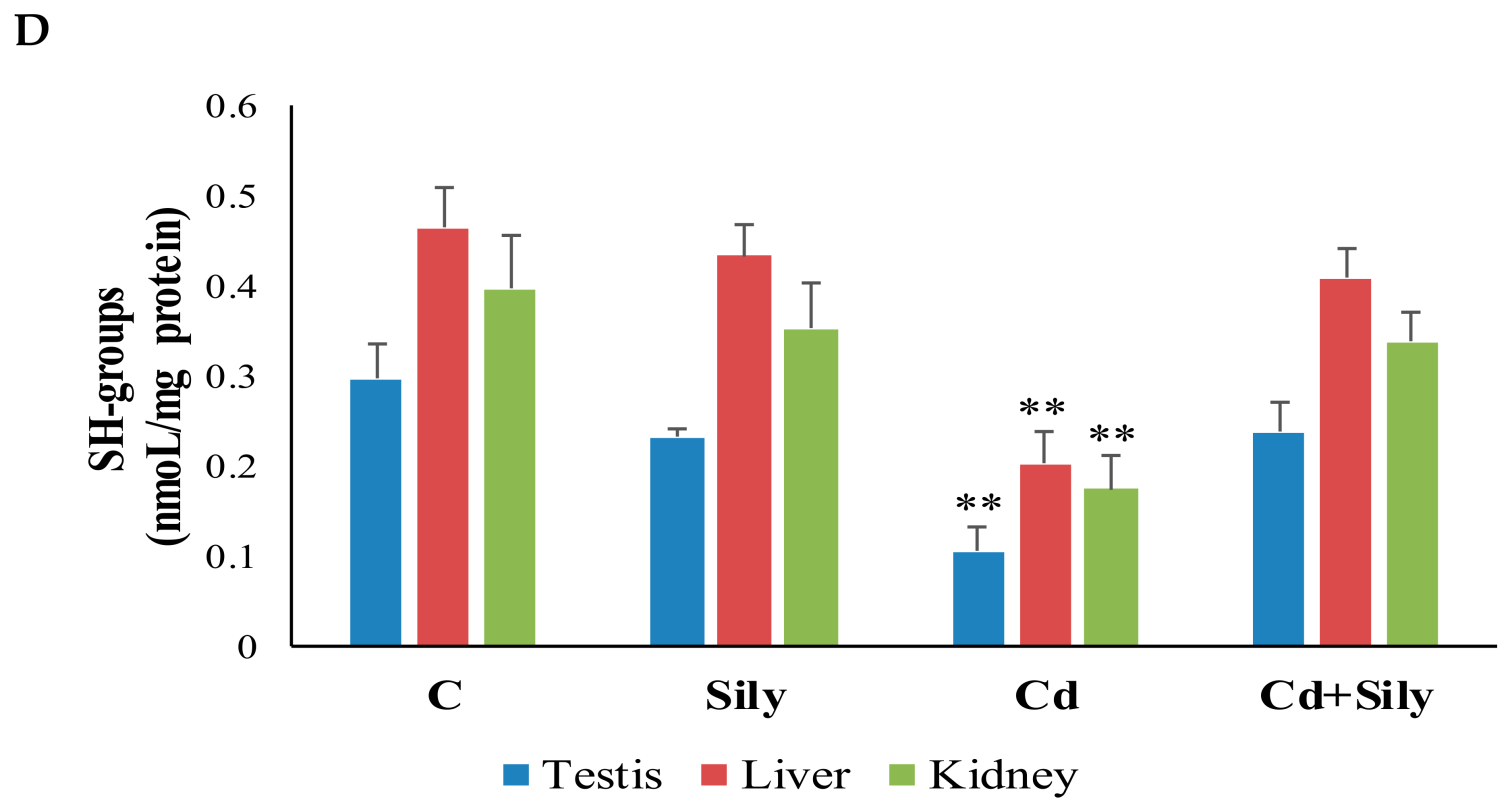
Effects of cadmium (5 mg/kg) and silymarin (30 mg/kg) on oxidative stress markers in testis, liver, and kidney tissues. (**A**) Malondialdehyde (MDA) levels (Testis, *p* = 0.001; Liver, 0.002, Kidney, *p* = 0.002); (**B**) Superoxide dismutase (SOD) activity (Testis, *p* = 0.003; Liver, 0.002, Kidney, *p* = 0.004), (**C**) Catalase (CAT) activity (Testis, *p* = 0.004; Liver, 0.005, Kidney, *p* = 0.002); (**D**) Sulfhydryl (-SH) group content (Testis, *p* = 0.006; Liver, 0.003, Kidney, *p* = 0.001). Data are expressed as mean ± SD (*n* = 6 per group). * *p* < 0.05, ** *p* < 0.01 vs. control group.

To evaluate the oxidative damage induced by cadmium and the potential protective effect of silymarin, lipid peroxidation (MDA), antioxidant enzyme activities (SOD and CAT), and sulfhydryl (-SH) group levels were measured in testicular, hepatic, and renal tissues (Figure 4). Rats exposed to cadmium chloride at a dose of 5 mg/kg bw (Cd group) showed a significant increase in MDA levels in all three organs compared to the control group, indicating elevated lipid peroxidation and oxidative stress. This increase was accompanied by a marked decrease in antioxidant defenses, as evidenced by a significant reduction in SOD and CAT enzymatic activities and SH group concentrations in the testis, liver, and kidney tissues. Interestingly, co-treatment with silymarin at a dose of 30 mg/kg bw (Cd + Sily group) significantly mitigated these cadmium-induced alterations. MDA levels were notably reduced in all organs compared to the Cd group, suggesting that lipid peroxidation was limited by the antioxidant properties of silymarin. Simultaneously, the activities of SOD and CAT enzymes, as well as the levels of SH groups, were significantly improved in silymarin co-treated rats, although they did not always return fully to control values. These findings suggest a partial restoration of the antioxidant balance in the presence of silymarin. In the Sily group, no significant differences were observed compared to the control group across all measured parameters, confirming the safety and antioxidant neutrality of silymarin when administered alone under physiological conditions.

### 3.9. Histological Analysis of Testicular, Liver and Kidney Tissues

#### 3.9.1. Histological Analysis of Testicular Tissue

**Figure 5:**
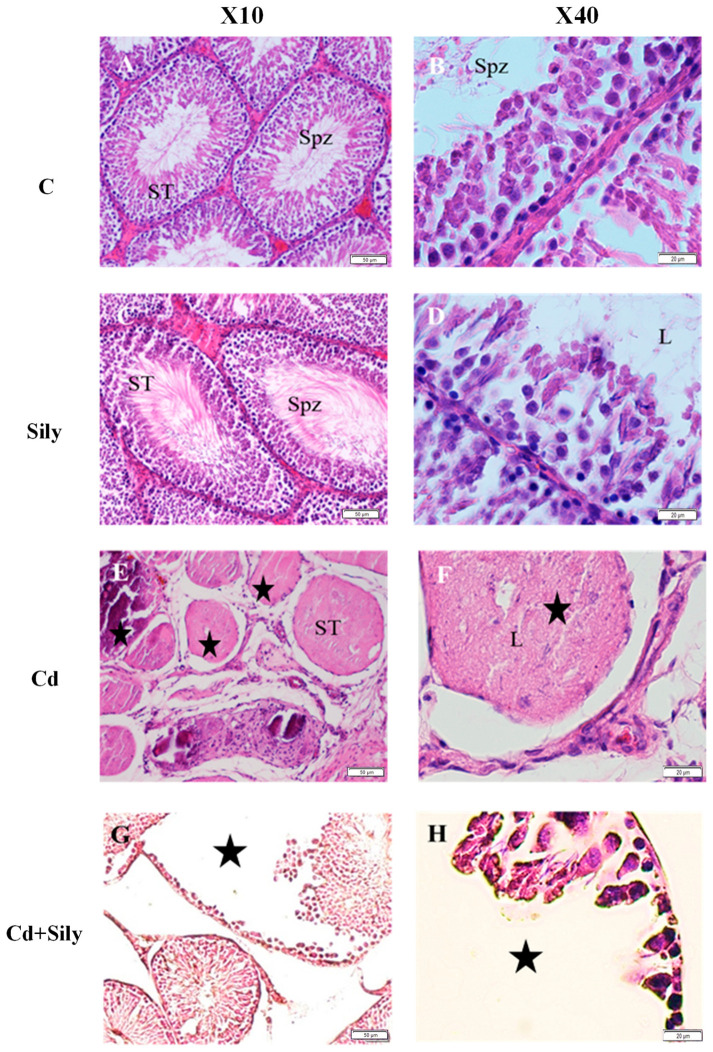
Histological sections of rat testes (H&E staining). Testis sections (**A**,**C**,**E**,**G**): magnification X10; Testis sections (**B**,**D**,**F**,**H**): magnification X40. (**A**,**B**) Control group (C) showing normal seminiferous tubules (ST) with organized germinal epithelium, lumen (L), and abundant spermatozoa (Spz). (**C**,**D**) Silymarin-treated group (Sily) displaying well-preserved seminiferous tubules (ST), intact germ cells, and normal spermatozoa (Spz) within the lumen (L). (**E**,**F**) Cadmium-exposed group (Cd) showing severe testicular alterations: disorganized seminiferous tubules (ST), loss of germ cells, and reduced spermatozoa (Spz) (stars). (**G**,**H**) Cadmium + silymarin co-treated group (Cd + Sily) exhibiting partial protection: seminiferous tubules (ST) showed a tendency toward reduced disorganization compared with the Cd group; however, the tubular architecture remained partially disrupted, with persistent loss of germ cells and spermatozoa (Spz) (stars) (Magnification ×10 and ×40 and scale bars are 50 µm and 20 µm, respectively).

Histological sections from control rats (Figure 5A,B) revealed normal testicular architecture with well-organized seminiferous tubules filled with abundant spermatozoa. All stages of germ cells, including spermatogonia, spermatocytes, and spermatids, were clearly observed, closely associated with Sertoli cells forming the seminiferous epithelium. The interstitial space appeared normal, with well-defined Leydig cells. In the Sily group (Figure 5C,D), the seminiferous tubules showed a preserved histological structure, similar to controls. All germ cell stages were present, and the luminal space remained densely packed with mature spermatozoa, suggesting no adverse effects of Silymarin on spermatogenesis or interstitial tissue.

Conversely, in the Cadmium-treated group (Cd) (Figure 5E,F), a marked depletion of germ cells was observed. The seminiferous tubules displayed an altered epithelium, with a noticeable absence of spermatozoa in the luminal space. The tubular architecture showed only limited preservation, with significant disruption of the germinal lineage. The interstitial tissue showed signs of vascular congestion and the presence of Leydig cells. In the group co-treated with Cadmium and Silymarin (Cd + Sily), partial recovery was observed (Figure 5G,H). While spermatozoa were still largely absent in the lumens, the seminiferous tubules exhibited a slight and inconsistent tendency toward reduced cellular disorganization compared to the Cd-only group, though this improvement remained modest and difficult to ascertain from histological images alone. However, proliferation of Leydig cells and peripheral fibrous connective tissue was noted around the tubules, indicating ongoing structural remodeling. These findings indicate that while Silymarin alone does not disrupt testicular histology, Cadmium exposure severely impairs spermatogenesis, and co-administration of Silymarin does not fully restore the germinal epithelium but may mitigate interstitial damage.

#### 3.9.2. Histopathological Evaluation of Testicular Damages

Table 6 shows the histopathological scoring of testicular injuries in the seminiferous tubules across the different experimental groups. All animals in the Control (C) and Silymarin-only (Sily) groups exhibited a score of 0, indicating normal histological architecture and confirming the absence of testicular toxicity associated with silymarin administration. In contrast, rats exposed to cadmium alone (Cd group) displayed pronounced testicular damage, with five out of six animals exhibiting severe degeneration (Score III) and one showing moderate lesions (Score II), underscoring the high gonadotoxic potential of cadmium. Co-administration of silymarin with cadmium (Cd + Sily group) led to a partial attenuation of testicular injury, as reflected by the presence of moderate lesions (Score II) in two animals. However, severe damage (Score III) persisted in the four rats (Table 6).

#### 3.9.3. Histopathological Evaluation of Kidney Damages

**Figure 6:**
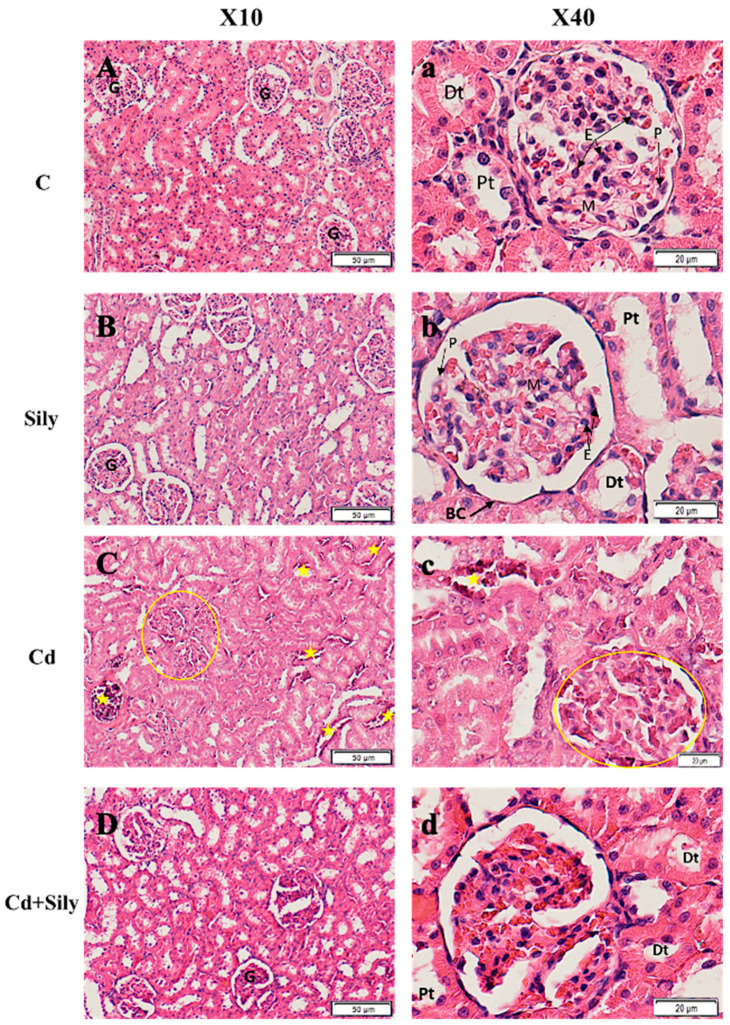
Histological observations of rat kidneys (H&E staining). Kidney sections (**A**–**D**): magnification ×10; Kidney sections a, b, c, and d: magnification ×40. (**A**,**a**) Control group (C) showing normal glomeruli (G) with intact Bowman capsules (BC) and regular proximal (Pt) and distal (Dt) tubular structures. (**B**,**b**) Silymarin-treated group (Sily) displaying glomerular (G) and tubular (Pt, Dt) architecture comparable to controls, with preserved epithelial cells (E) and mesangial cells (M). (**C**,**c**) Cadmium-exposed group (Cd) showing marked nephrotoxicity: glomerular disorganization, congested capillaries (arrows), dilated Bowman’s spaces (circled areas), tubular degeneration, cytoplasmic vacuolization (stars), and interstitial vascular congestion. (**D**,**d**) Cadmium + Silymarin co-treated group (Cd + Sily) exhibiting partial improvement: better-preserved glomeruli (circled), attenuated tubular lesions, reduced vacuolization (stars), and less interstitial congestion (Magnification ×10 and ×40 and scale bars are 50 µm and 20 µm, respectively).

Histological examination of kidney sections (Figure 6) of control rats revealed normal renal architecture, with well-preserved glomeruli and intact Bowman’s capsules, and regularly arranged tubules with normal epithelial lining and lumens (Figure 6A,a). Similarly, the silymarin-treated group (Sily) showed a histological appearance comparable to controls, with no detectable pathological changes (Figure 6B,b). In cadmium-exposed rats (Cd), pronounced structural alterations were observed, including glomerular disorganization, congested capillaries, dilated Bowman’s spaces (circled areas), tubular degeneration, luminal dilation, loss of the brush border, and interstitial vascular congestion (Figure 6C,c), consistent with nephrotoxicity and oxidative imbalance. In the cadmium + Silymarin co-treated group (Cd + Sily), renal histology showed partial improvement, with relatively preserved glomerular architecture, attenuated tubular lesions, improved epithelial integrity, and reduced interstitial alterations, although mild degenerative changes persisted (Figure 6D,d).

#### 3.9.4. Histopathological Evaluation of Liver Damages

**Figure 7:**
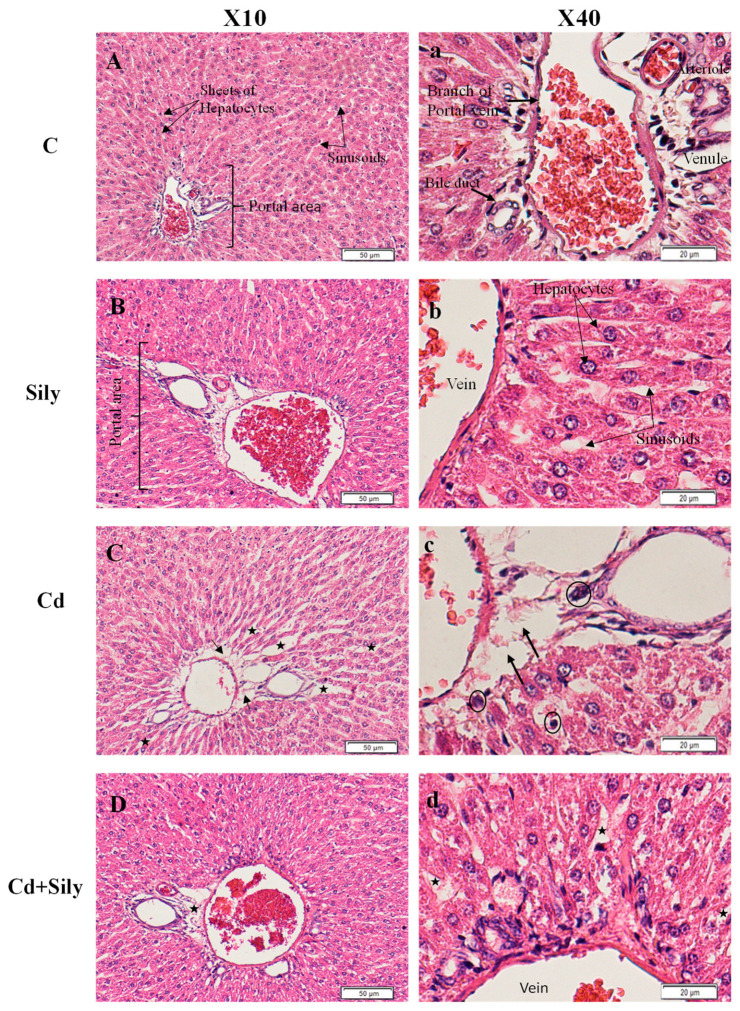
Histological observations of rat liver sections (H&E staining). Liver sections (**A**–**D**): magnification ×10; Liver sections a, b, c and d: magnification ×40. (**A**,**a**) Control group (C) showing normal hepatic architecture with well-organized cords of hepatocytes radiating from the central vein, regular sinusoids, and intact portal areas containing branches of the portal vein, hepatic artery, and bile duct. (**B**,**b**) Silymarin-treated group (Sily) exhibiting hepatic histology comparable to controls, with preserved hepatocyte morphology, normal sinusoidal organization, and well-defined vascular and biliary structures. (**C**,**c**) Cadmium-exposed group (Cd) displaying marked hepatic injury, characterized by disorganization of hepatocyte cords, pronounced sinusoidal dilatation and congestion (stars), and hepatocytes showing severe degenerative changes (circled areas), reflecting significant hepatic stress. (**D**,**d**) Cadmium + silymarin co-treated group (Cd + Sily) showing partial improvement of hepatic architecture, with improved hepatocyte organization, reduced sinusoidal dilatation (stars), and attenuation of degenerative changes, although mild alterations persist (Magnification ×10 and ×40 and scale bars are 50 µm and 20 µm, respectively).

Histological examination of rat liver sections revealed distinct architectural changes across the experimental groups (Figure 7). In the control group, liver sections displayed normal hepatic architecture characterized by well-organized hepatocyte cords radiating from the central vein, regular sinusoids, and intact portal areas (Figure 7A). Similarly, the silymarin-treated group showed preserved hepatic histology comparable to controls, with normal hepatocyte morphology and well-defined vascular and biliary structures (Figure 7B). In contrast, cadmium-exposed rats exhibited marked hepatic injury, including disorganization of hepatocyte cords, pronounced sinusoidal dilatation and congestion, as well as severe degenerative changes in hepatocytes, reflecting significant hepatic stress (Figure 7C). Notably, co-treatment with silymarin partially attenuated cadmium-induced hepatic damage, as evidenced by improved hepatocyte organization, reduced sinusoidal dilatation, and a decrease in degenerative changes, although mild alterations remained visible (Figure 7D).

### 3.10. Molecular Docking Studies

Molecular docking simulations were performed to investigate the binding affinity and interaction profile of silybin, a major bioactive compound of *Silybum marianum*, with key antioxidant enzymes: catalase (CAT), superoxide dismutase (SOD), and glutathione peroxidase (GPx). The docking analysis was conducted using AutoDock Vina, and the results are summarized in Table 7.

Silybin exhibited the most favorable binding energy with GPx (−8.4 kcal/mol), followed closely by CAT (−8.3 kcal/mol), and showed a slightly weaker interaction with SOD (−6.4 kcal/mol). These values suggest a favorable computational affinity of silybin for antioxidant enzymes, providing a preliminary mechanistic hypothesis that warrants further experimental validation.

**Figure 8:**
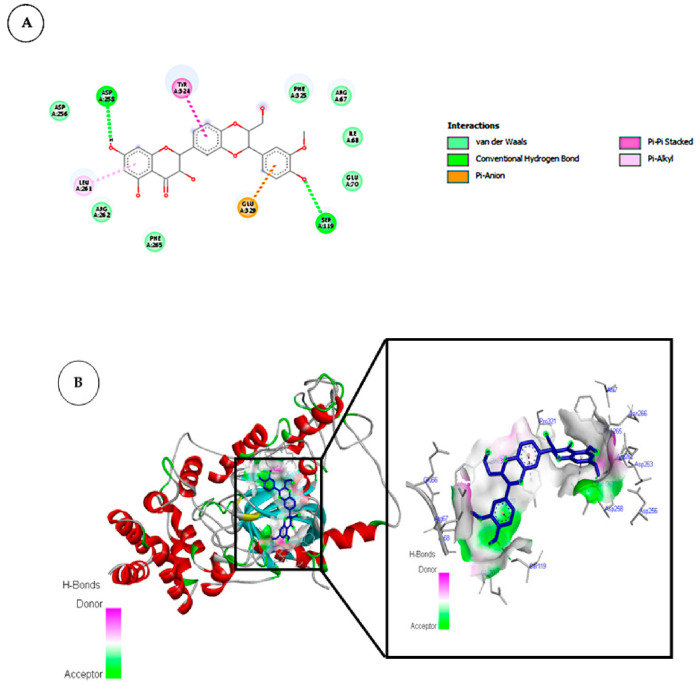
Two-dimensional (**A**) and the three-dimensional (**B**) representations of the molecular docking interaction between Silybin and the binding site of catalase (CAT) (PDB ID: 4MCM). The docking conformation highlights key residues involved in stabilizing the complex.

In the CAT–silybin complex, two conventional hydrogen bonds were formed, and key interacting amino acid residues included ASP256, ASP258, TYR324, PHE325, ARG67, ILE68, GLU70, SER119, GLU329, PHE265, ARG262, and LEU261. These residues are located in or near the enzyme’s active site, contributing to binding stability through hydrogen bonding, van der Waals forces, and electrostatic interactions (Figure 8).

**Figure 9:**
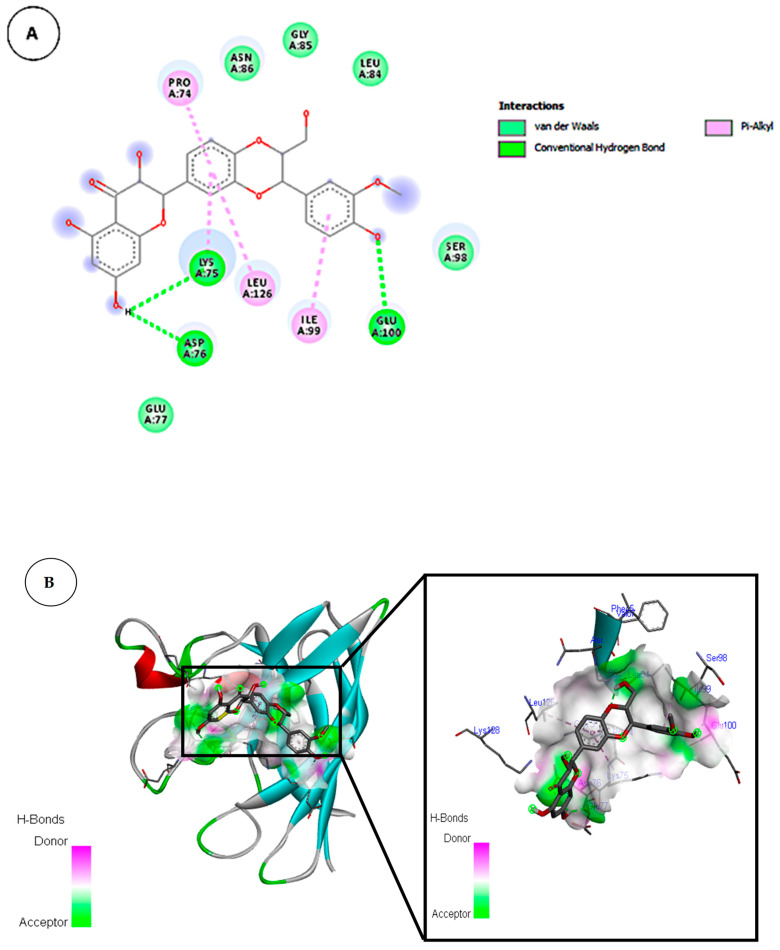
The two-dimensional (**A**) and the three-dimensional (**B**) representations of the docking interaction between the silybin molecule and the active site of superoxide dismutase (SOD) (PDB ID: 1DGB). The binding conformation illustrates key intermolecular interactions contributing to complex stability.

As shown in Figure 9, the SOD–silybin interaction involved three hydrogen bonds with amino acids PRO74, ASN86, and GLY85, in addition to hydrophobic and polar contacts with LEU84, SER98, GLU100, ILE99, LEU126, LYS75, and ASP76. These intermolecular interactions are essential for complex stabilization despite the relatively lower binding energy.

**Figure 10:**
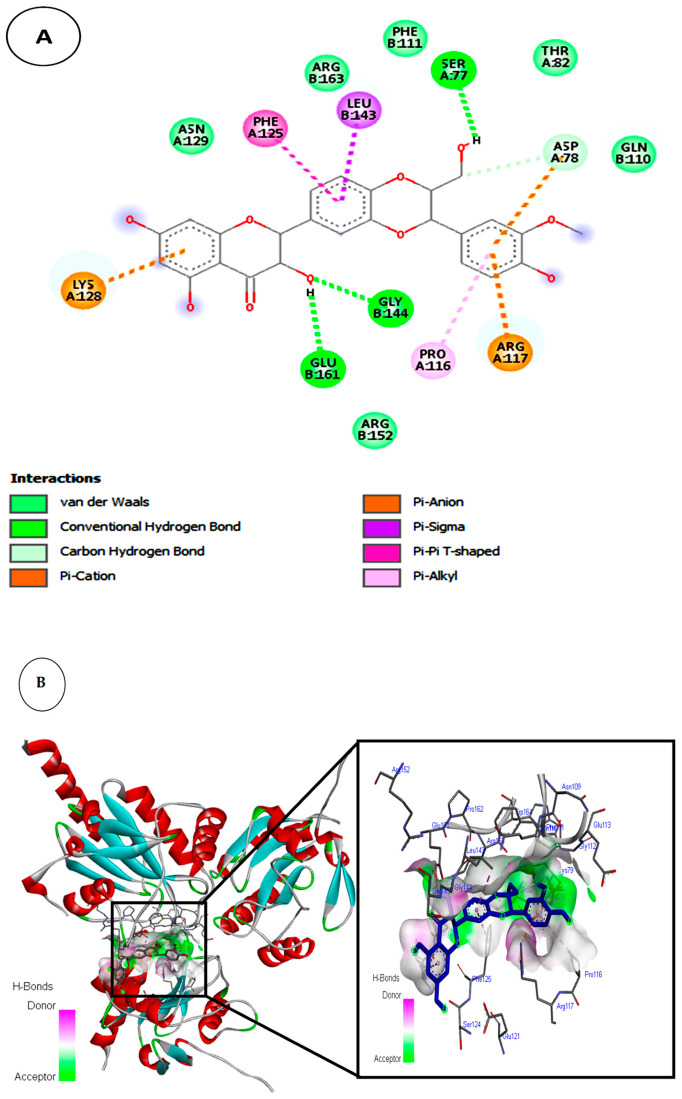
The two-dimensional (**A**) and the three-dimensional (**B**) representations of the docking interaction between the silybin molecule and the active site of glutathione peroxidase (GPx) (PDB ID: 1DGB). The binding conformation illustrates key intermolecular interactions contributing to complex stability.

The GPx–silybin complex, illustrated in Figure 10, demonstrated a strong binding profile with three conventional hydrogen bonds and numerous van der Waals interactions. Residues involved included ASN129, PHE125, LEU143, ARG163, PHE111, SER77, THR82, ASP78, GLN110, ARG117, PRO116, GLY144, GLU161, ARG152, and LYS128, reflecting a rich network of stabilizing interactions.

## 4. Discussion

### 4.1. Systemic and Metabolic Effects of Cadmium: Role of Oxidative Stress

Subchronic exposure to cadmium chloride (5 mg/kg bw for six weeks) resulted in a significant reduction in body weight compared to control animals. This finding is consistent with previous studies describing body weight loss as a characteristic systemic manifestation of cadmium toxicity [39,40]. Cadmium is known to disrupt energy homeostasis through multiple mechanisms, including the induction of oxidative stress, alteration of appetite-regulating hormones, and interference with digestive enzyme activity, ultimately leading to reduced food intake and impaired nutrient utilization [39,41]. In addition, cadmium-induced gastrointestinal mucosal damage may compromise nutrient absorption, while hepatic and pancreatic dysfunction can further impair protein synthesis and carbohydrate metabolism, collectively contributing to body weight decline.

Co-administration of silymarin at a dose of 30 mg/kg did not prevent cadmium-induced weight loss. Although silymarin possesses well-established antioxidant and hepatoprotective properties [42], its protective efficacy against cadmium-induced metabolic disturbances appears limited under the present experimental conditions. This lack of effect may be related to the administered dose or treatment duration, which may not have been sufficient to counteract the profound systemic metabolic alterations induced by cadmium exposure. Furthermore, previous reports have suggested that high doses of silymarin may exert mild anorexigenic effects, which could partially contribute to the inability to restore body weight in cadmium-exposed rats [43,44].

Cadmium exposure also markedly affected hematological and immune parameters, reflecting its systemic immunotoxic effects. Consistent with earlier findings, reductions in lymphocytes, monocytes, neutrophils, and eosinophils were observed, suggesting impaired hematopoiesis, increased immune cell apoptosis, or suppression of bone marrow progenitor proliferation [45]. Such alterations may compromise both innate and adaptive immune defenses, increasing susceptibility to infections and contributing to inflammatory dysregulation.

Partial normalization of certain immune parameters in the cadmium plus silymarin group indicates a modest cytoprotective effect of silymarin, potentially mediated through enhancement of antioxidant defenses and attenuation of oxidative stress in hematopoietic tissues. Silymarin has been reported to upregulate endogenous antioxidant systems, including glutathione, superoxide dismutase, and catalase, thereby limiting oxidative damage to immune cells [46]. The increase in hemoglobin levels observed following cadmium exposure may represent a compensatory response involving altered erythropoietin signaling or changes in plasma volume [47]. The further elevation of hemoglobin in the cadmium plus silymarin group could suggest a stimulatory effect on erythropoiesis, although this hypothesis requires further investigation.

Alterations in lipid metabolism were also evident following cadmium exposure, as shown by increased triglyceride and HDL cholesterol levels. These disturbances are consistent with previous reports and may result from cadmium-induced inhibition of lipoprotein lipase activity, impaired lipid clearance, and hepatic dysfunction [48]. The increase in HDL cholesterol may reflect a compensatory response to oxidative stress or disrupted hepatic and endothelial lipid handling [49]. Silymarin treatment did not normalize these lipid abnormalities, suggesting that persistent oxidative and inflammatory stress induced by cadmium may exceed its antioxidant capacity under the present experimental conditions.

### 4.2. Integrated Multi-Organ Toxicity of Cadmium: Hepatic, Renal and Reproductive Damage

The hepatic, renal, and reproductive alterations observed in the present study should be interpreted as interconnected manifestations of cadmium-induced systemic toxicity rather than isolated organ-specific effects.

Subchronic cadmium exposure induced marked hepato-renal toxicity, as evidenced by significant alterations in liver and kidney function biomarkers. In the liver, cadmium-treated rats exhibited significant increases in serum alanine aminotransferase (ALT), aspartate aminotransferase (AST), and bilirubin levels, indicating hepatocellular injury and impaired hepatic function. These enzymes are widely recognized as sensitive indicators of membrane integrity loss and hepatocyte necrosis [50]. Elevated bilirubin levels further suggest compromised hepatic clearance capacity or enhanced hemolysis exceeding hepatic processing ability [51].

The hepatotoxic effects of cadmium are primarily mediated through excessive reactive oxygen species (ROS) generation, mitochondrial dysfunction, and activation of inflammatory pathways, ultimately leading to hepatocellular degeneration and necrosis [52]. Cadmium can also disrupt metal homeostasis and impair mitochondrial respiratory complexes, exacerbating oxidative stress and energy failure within hepatocytes.

Despite the well-documented hepatoprotective properties of silymarin, co-treatment at 30 mg/kg did not significantly attenuate the cadmium-induced elevation of hepatic biomarkers. This limited efficacy may be attributed to the severity of cadmium-induced liver injury or to the inability of silymarin, at the administered dose and duration, to counteract cadmium-specific mechanisms such as metal accumulation and mitochondrial damage. Previous studies have shown that silymarin is particularly effective against hepatotoxins that predominantly induce oxidative stress; however, cadmium toxicity involves additional pathways that may not be fully addressed by antioxidant therapy alone [53].

Renal function was also markedly impaired following cadmium exposure, as demonstrated by a significant increase in serum creatinine levels, reflecting reduced glomerular filtration and tubular dysfunction. Cadmium nephrotoxicity is well established and involves preferential accumulation in proximal tubular cells, where it induces oxidative stress, inflammation, mitochondrial injury, and apoptosis [54]. These mechanisms collectively contribute to progressive renal dysfunction, as observed in the present study.

Silymarin administration failed to prevent the cadmium-induced increase in creatinine levels, suggesting limited nephroprotective efficacy under the present experimental conditions. This may be related to suboptimal renal bioavailability following oral administration or insufficient tissue concentrations at the administered dose [55]. Moreover, the complex and multifactorial nature of cadmium-induced renal injury, including impaired repair mechanisms and persistent mitochondrial dysfunction, may limit the effectiveness of silymarin when used as a monotherapy. These findings indicate that higher doses, prolonged treatment, or combination strategies may be required to achieve meaningful reno-protection in the context of PTE toxicity. Within this context of sustained systemic oxidative stress, the male reproductive system appears particularly vulnerable to cadmium exposure, given its high metabolic activity and dependence on redox homeostasis.

Hepatic dysfunction may aggravate systemic oxidative stress and metabolic imbalance, while renal impairment may reduce cadmium elimination, thus increasing tissue damage and contributing to reproductive toxicity.

Subchronic exposure to cadmium induced marked reproductive toxicity in male rats, as demonstrated by significant impairments in sperm count, motility, and morphology assessed by computer-assisted sperm analysis (CASA). These alterations are consistent with previous reports identifying cadmium as a potent reproductive toxicant capable of compromising male fertility even at relatively low exposure levels [56]. The deleterious effects of cadmium on sperm quality are primarily mediated through excessive oxidative stress, endocrine disruption, and direct cytotoxicity to the germinal epithelium [57]. Oxidative stress plays a central role in cadmium-induced spermatogenic damage. Cadmium promotes the generation of reactive oxygen species (ROS) within testicular tissue while simultaneously impairing endogenous antioxidant defenses, leading to lipid peroxidation of sperm membranes, DNA damage, and loss of sperm motility. In addition, cadmium interferes with calcium homeostasis by inhibiting calcium channels essential for sperm flagellar movement and acrosome reaction [58]. The decrease in serum calcium levels observed in cadmium-exposed rats supports this mechanism and likely contributes to the pronounced asthenozoospermia detected in this study. Although the exposure duration did not encompass a complete spermatogenic cycle, it was sufficient to induce significant cadmium-related spermatogenic impairment and to evaluate the protective efficacy of silymarin against early and intermediate testicular damage.

Cadmium also disrupts the hypothalamic–pituitary–gonadal (HPG) axis by inducing apoptosis of Sertoli and Leydig cells, leading to impaired testosterone synthesis and defective spermatogenesis [59]. Histological examination confirmed these effects, revealing seminiferous tubule degeneration, germ cell loss, and disorganization of the spermatogenic epithelium. Although the six-week exposure period did not fully encompass the entire spermatogenic cycle, including epididymal transit and sperm maturation, it was sufficient to induce significant cadmium-related spermatogenic impairment. Consequently, the observed sperm parameters may not reflect the full cumulative impact of cadmium exposure, and the protective effects of silymarin on spermatogenesis may have been partially underestimated.

In contrast to previous studies reporting beneficial effects of silymarin on cadmium- or potentially toxic elements-induced reproductive damage [23,60], silymarin administration at 30 mg/kg did not significantly improve sperm parameters or fully restore testicular histoarchitecture in the present study. This discrepancy may be attributed to differences in dosage, duration of treatment, formulation, or route of administration. Furthermore, cadmium-induced reproductive toxicity involves complex endocrine and transcriptional dysregulation within the HPG axis, which may not be fully corrected by antioxidant therapy alone [61].

Although a complete recovery of the seminiferous tubule structure was not observed in the Cd + silymarin group. This partial effect may reflect a limited capacity of silymarin to protect the germ cell niche under conditions of sustained cadmium exposure. The absence of serum hormone measurements, particularly testosterone, represents a limitation of this study and precludes a more comprehensive assessment of HPG axis involvement. Future studies incorporating endocrine profiling and extended treatment durations are warranted to better clarify the reproductive protective potential of silymarin. These functional and histological impairments further support the involvement of oxidative stress-mediated mechanisms, providing a rationale for exploring the molecular antioxidant targets of silybin.

### 4.3. Protective Effects of Silymarin Against Cadmium-Induced Systemic Toxicity: Biochemical, Histological and Molecular Docking Insights

Given the systemic nature of cadmium toxicity, the protective effects of silymarin were evaluated across multiple organs rather than within a single target tissue.

Systemic oxidative stress represents a central mechanism underlying cadmium-induced multi-organ toxicity. Cadmium exposure enhances the generation of reactive oxygen species (ROS) while simultaneously depleting endogenous antioxidant defenses, thereby creating a redox imbalance that affects multiple tissues, including the liver, kidneys, immune system, and testes. This pro-oxidant environment promotes lipid peroxidation, protein oxidation, mitochondrial dysfunction, and activation of apoptotic pathways, ultimately leading to organ dysfunction and structural damage [5,52].

In the present study, cadmium-induced oxidative stress was indirectly reflected by the widespread biochemical and functional alterations observed across several organ systems. The partial normalization of immune parameters in the cadmium plus silymarin group supports the hypothesis that silymarin exerts a systemic antioxidant effect, likely by reinforcing cellular redox defenses. Silymarin is known to enhance intracellular glutathione levels and to upregulate the activity of key antioxidant enzymes, including superoxide dismutase (SOD), catalase (CAT), and glutathione peroxidase (GPx), thereby limiting ROS-mediated cellular injury [46]. It should be noted that the absence of significant changes in oxidative stress markers in the silymarin-only group compared to controls suggests that silymarin does not enhance antioxidant status under physiological conditions, but rather exerts its effects primarily under conditions of oxidative stress.

Docking is now presented as a complementary analysis, providing additional support to the experimental data, but not as confirmatory or definitive evidence of the proposed antioxidant mechanisms. It should be noted, however, that molecular docking simulations do not account for protein flexibility, solvent effects, or intracellular bioavailability, and therefore these results should be interpreted as supportive computational evidence rather than confirmatory proof of direct enzyme modulation. Silybin, the major bioactive constituent of silymarin, exhibited strong binding affinity toward CAT, SOD, and GPx, with binding energies ranging from −6.4 to −8.4 kcal/mol. These stable interactions, mediated by multiple hydrogen bonds and hydrophobic contacts with critical active-site residues, suggest that silybin may stabilize the active conformations of these enzymes and potentially enhance their catalytic efficiency. Such interactions could facilitate more effective detoxification of ROS, thereby mitigating oxidative stress at the cellular level.

These findings are consistent with previous reports demonstrating that silybin directly scavenges ROS, preserves mitochondrial integrity, and prevents oxidative damage in various experimental models [62,63]. In particular, silybin has been shown to inhibit mitochondrial ROS production, reduce lipid peroxidation, and promote mitochondrial biogenesis, mechanisms that are highly relevant in the context of cadmium-induced mitochondrial dysfunction. Nevertheless, despite the favorable antioxidant profile of silybin suggested by both experimental and in silico data, silymarin treatment did not fully reverse the histological and biochemical damage induced by cadmium in the liver, kidneys, and testes. This apparent discrepancy underscores the complexity and severity of cadmium toxicity, which involves not only oxidative stress but also heavy metal accumulation, mitochondrial injury, endocrine disruption, and impaired cellular repair mechanisms. These processes may exceed the protective capacity of antioxidant therapy alone, particularly at the administered dose and exposure duration. Notably, co-administration of silymarin did not ameliorate cadmium-induced body weight loss, which remained comparable between the Cd and Cd + Sily groups. This apparent discrepancy with the partial protective effects observed at the biochemical and histological levels may be explained by the multifactorial nature of cadmium-induced weight loss, which involves not only oxidative stress but also anorexia, intestinal malabsorption, muscle catabolism, and metabolic disruption. These systemic mechanisms are unlikely to be fully counteracted by silymarin’s antioxidant properties alone, particularly at the administered dose and over the six-week exposure period. Furthermore, cadmium accumulates progressively in tissues and may exert sustained metabolic effects that exceed the protective capacity of silymarin monotherapy. This observation further supports the conclusion that silymarin confers organ-specific rather than global systemic protection under subchronic cadmium exposure conditions, and highlights the need for combination strategies targeting multiple toxicity pathways simultaneously.

Overall, the integration of biochemical and histological data, supported by preliminary computational evidence from molecular docking, suggests a possible role of silybin in modulating key redox pathways, pending further experimental confirmation. However, the incomplete protection observed in vivo suggests that prolonged administration, higher dosing, or combination strategies with metal chelators or complementary antioxidants may be necessary to achieve optimal tissue protection under subchronic cadmium exposure conditions.

A limitation of the present study is the absence of direct mechanistic investigations, such as ROS quantification and the evaluation of key signaling pathways including Nrf2 and NF-κB, which would have provided deeper insight into the molecular mechanisms underlying the observed effects.

## 5. Conclusions

Subchronic cadmium exposure induced pronounced reproductive, hepatic, renal, and metabolic toxicity in adult male rats, as evidenced by impaired sperm quality, testicular histopathological damage, dyslipidemia, altered liver and kidney function markers, and significant changes in organ weights. Silymarin co-administration exerted partial but significant protective effects by attenuating oxidative stress, partially restoring antioxidant enzyme activities, and reducing histopathological damage in hepatic and renal tissues. However, these beneficial effects remained incomplete, particularly at the testicular level, where spermatogenesis was not fully restored, highlighting the limitations of silymarin monotherapy under conditions of sustained PTE exposure. These findings should be interpreted with caution, given the small sample size and the absence of direct mechanistic investigations such as ROS quantification and Nrf2/NF-κB pathway evaluation. The molecular docking results, while providing supportive computational insight into silybin–antioxidant enzyme interactions, require further experimental validation. Future studies should focus on optimizing silymarin dosing regimens and treatment durations, exploring combination strategies with metal chelators or Nrf2-activating agents, and investigating its effects in female models and chronic exposure scenarios. The use of commercially standardized silymarin formulations, as adopted in the present study, further supports its translational relevance and warrants clinical investigation in populations at risk of PTE exposure.

## Figures and Tables

**Table 1:** Physicochemical Characteristics and Composition of Silymarin Capsules (NOW^®^ Brand).

Parameter	Description
Commercial Name	Silymarin 150 mg—NOW^®^
Pharmaceutical Form	Capsules (Vcaps^®^—vegetarian capsules)
Active Ingredient	Milk Thistle Extract (*Silybum marianum*) standardized to contain silymarin
Silymarin Content	150 mg per capsule
Additional Components	Cellulose (capsule), magnesium stearate (vegetable source), silica
Recommended Use	Liver support, antioxidant protection
Presentation	Bottle containing 120 capsules
Mode of Administration	Oral
Storage Conditions	Store in a cool, dry place after opening
Manufacturer	NOW Foods, Bloomingdale, Illinois, IL, USA
Certifications	GMP Quality Assured, Non-GMO, Vegan/Vegetarian

**Table 2:** Effect of chronic treatment with Silymarin (30 mg/kg) and Cadmium (5 mg/kg) on body weight (bw) in rats. Results are expressed as the mean ± SD (*n* = 6). The star (*) indicates a significant difference (*p* < 0.05) compared with the control (C).

Group	Initial Weight (g)	Final Weight (g)	% Change
C	315 ± 10	431 ± 12	+36.8%
Sily	305 ± 11	438 ± 14	+43.6%
Cd	325 ± 13	225 ± 15 * p = 0.03	−30.8%
Cd + Sily	310 ± 10	210 ± 13 * p = 0.02	−32.3%

**Table 3:** Relative organ weight following chronic treatment with Silymarin (30 mg/kg) and Cadmium (5 mg/kg) in rats. The results are presented as the mean ± SD (*n* = 6). The stars (*) and (**) indicate significant differences compared with the control (C) group at *p* < 0.05 and *p* < 0.01, respectively.

Relative Organ Weight (g/100 g bw)								
Groups	Testis	Epididymis	Prostate	Seminal Vesicles	Liver	Kidney	Heart	Brain
**C**	0.42 ± 0.02	0.31 ± 0.02	0.31 ± 0.03	0.68 ± 0.03	3.95 ± 0.18	0.38 ± 0.02	0.40 ± 0.04	0.50 ± 0.06
**Sily**	0.43 ± 0.04	0.33 ± 0.03	0.32 ± 0.02	0.75 ± 0.02	4.02 ± 0.01	0.36 ± 0.02	0.42 ± 0.03	0.49 ± 0.04
**Cd**	0.15 ± 0.02 ** *p* = 0.001	0.22 ± 0.02 ** *p* = 0.004	0.18 ± 0.02 ** *p* = 0.002	0.45 ± 0.03 ** *p* = 0.004	2.50 ± 0.01 * *p* = 0.03	0.25 ± 0.02 * *p* = 0.03	0.35 ± 0.05	0.42 ± 0.02 * *p* = 0.04
**Cd+ Sily**	0.19 ± 0.02 ** *p* = 0.002	0.25 ± 0.02 ** *p* = 0.005	0.20 ± 0.02 ** *p* = 0.002	0.50 ± 0.02 ** *p* = 0.003	2.60 ± 0.03 * *p* = 0.04	0.30 ± 0.03 * *p* = 0.04	0.34 ± 0.04	0.47 ± 0.01

**Table 4:** Effects of Cadmium and Silymarin on Hematological Parameters. The results are presented as the mean ± SD (*n* = 6). The stars (*) and (**) indicate significant differences compared with the control (C) group at *p* < 0.05 and *p* < 0.01, respectively.

Parameter	C	Sily	Cd	Cd + Sily
Lymphocytes (10^3^/µL)	3.50 ± 0.05	3.65 ± 0.05	2.06 ± 0.05 * p = 0.02	2.92 ± 0.05 * p = 0.04
Monocytes (10^3^/µL)	0.24 ± 0.002	0.28 ± 0.022	0.19 ± 0.002 * p = 0.02	0.26 ± 0.003
Eosinophils (10^3^/µL)	0.35 ± 0.006	0.34 ± 0.005	0.22 ± 0.004 * p = 0.02	0.39 ± 0.007 * p = 0.03
Neutrophils (10^3^/µL)	1.66 ± 0.002	1.53 ± 0.022	0.98 ± 0.016 * p = 0.03	1.03 ± 0.017 ** p = 0.001
Basophils (10^2^/µL)	0.047 ± 0.008	0.049 ± 0.008	0.039 ± 0.001	0.035 ± 0.010
HEMOGLOBIN (g/dL)	13.32 ± 1.54	12.06 ± 1.51	13.96 ± 1.91 * p = 0.04	14.56 ± 0.69 ** p = 0.004
Total Ig (10^3^/µL)	0.030 ± 0.004	0.027 ± 0005	0.030 ± 0.003	0.027 ± 0.005

**Table 5:** Effect of Silymarin on serum lipid profile in cadmium-exposed rats. The results are expressed as the mean ± SD (*n* = 6) in mmoL/L. The stars (**) indicate significant differences compared with the control (C) group at *p* < 0.01.

Parameter	C	Sily	Cd	Cd + Sily
Total Cholesterol (TC)	0.68 ± 0.03	0.7 ± 0.04	1.23 ± 0.04 ** p = 0.003	0.93 ± 0.05 ** p = 0.005
LDL-C	0.75 ± 0.03	0.79 ± 0.03	1.05 ± 0.04 ** p = 0.003	0.99 ± 0.02 ** p = 0.007
HDL-C	0.95 ± 0.04	0.89 ± 0.02	0.65 ± 0.03 ** p = 0.004	0.69 ± 0.03 ** p = 0.003
Triglycerides (TG)	0.62 ± 0.03	0.59 ± 0.01	1.08 ± 0.06 ** p = 0.007	0.94 ± 0.04 ** p = 0.005

**Table 6:** Histopathological assessment of testicular injury scores in seminiferous tubules of rats subjected to cadmium and/or silymarin treatment.

GROUPS (*n* = 6)	SCORE 0	SCORE I	SCORE II	SCORE III
C	6	0	0	0
Sily	6	0	0	0
Cd	0	0	1 * p = 0.02	5 * p = 0.03
Cd + Sily	0	0	2 * p = 0.04	4 * p = 0.02

Testicular damage was graded on a four-point scale: Score 0 = normal histological architecture; Score I = mild degeneration; Score II = moderate degeneration; Score III = severe degeneration of seminiferous tubules. No histopathological abnormalities were observed in the control and silymarin-only groups (Score 0 in all animals). In contrast, cadmium exposure induced marked testicular damage, with most animals exhibiting severe degeneration (Score III). Co-administration of silymarin with cadmium led to a partial attenuation of testicular damage, as reflected by a redistribution of injury scores from predominantly severe (Score III) to moderate lesions (Score II) in a subset of animals. Data are expressed as mean ± SD for six rats each: *: *p* < 0.05 vs. control group (C).

**Table 7:** Docking energy values, the number of conventional hydrogen bonds, and the specific binding site residues involved in the interactions between Silybin and the target proteins CAT, SOD and GPx were analyzed using AutoDock Vina.

	Intermolecular Interactions		
Protein	Binding Energy (kcal/mol)	H-Bonds	Binding Amino Acid Residues
CAT	−8.3	2	ASP256, ASP258, TYR324, PHE325, ARG67, ILE68, GLU70, SER119, GLU329, PHE265, ARG262, and LEU261.
SOD	−6.4	3	PRO74, ASN86, GLY85, LEU84, SER98, GLU100, ILE99, LEU126, LYS75, and ASP76.
GPx	−8.4	3	ASN129, PHE125, LEU143, ARG163, PHE111, SER77, THR 82, ASP78, GLN110, ARG117, PRO116, GLY144, GLU161, ARG152, and LYS128.

**Figure A1:**
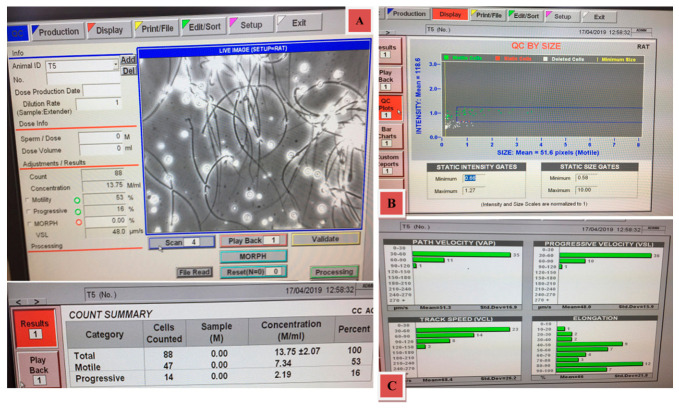
Evaluation of Sperm Quality in Control Rat Using CASA Software (Computer-Assisted Sperm Analysis). (**A**): Live image capture and sperm motility assessment using CASA software. Key parameters recorded include total concentration (13.75 M/mL), motility (53%), and progressive motility (16%), with a progressive velocity (VSL) of 49.0 μm/s. (**B**): Quality control graph by sperm size and intensity. This plot allows the exclusion of debris or non-sperm particles by applying static intensity and size gates. Motile cells are represented within defined thresholds. (**C**): Kinematic parameters of sperm motility, including: VAP (Path Velocity): Average velocity over a smoothed path (35 μm/s); VSL (Straight-line Velocity): Velocity along a straight line between the first and last detected point (36 μm/s); VCL (Track Speed): Total track velocity (48 μm/s); Elongation: Sperm head shape indicator (mean: 53%).

**Figure A2:**
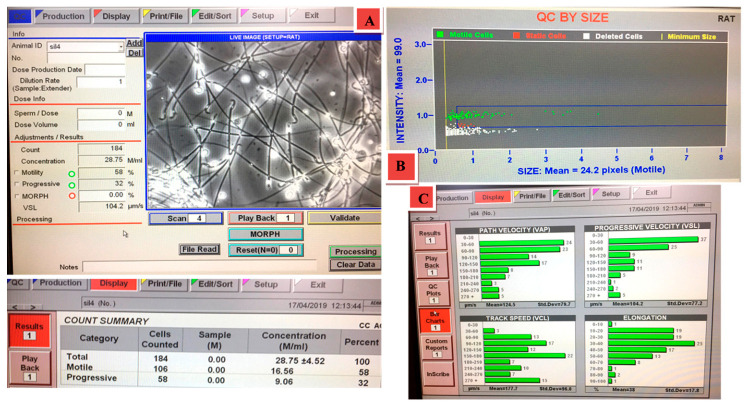
Sperm Quality Evaluation in Rat Treated with Silymarin (30 mg/kg) Using CASA Software. (**A**): CASA live capture and sperm tracking for a rat treated with silymarin. The total sperm concentration was **28.75 M/mL**, with a motility rate of **58%** and a progressive motility of **32%**. These values indicate enhanced sperm parameters compared to the control. (**B**): Size and intensity distribution of detected spermatozoa. The motile sperm population (green dots) is largely within the defined quality control thresholds for intensity and pixel size, supporting the accuracy of tracking. (**C**): Detailed kinematic sperm parameters: VAP (Path Velocity): 23.6 μm/s; VSL (Straight-line Velocity): 22.7 μm/s; VCL (Curvilinear Velocity): 27.9 μm/s; Elongation: 58%, indicating a more elongated and morphologically normal head shape. The overall data suggest that silymarin administration has similar sperm concentration, motility, and kinetic profiles to the untreated control group.

**Figure A3:**
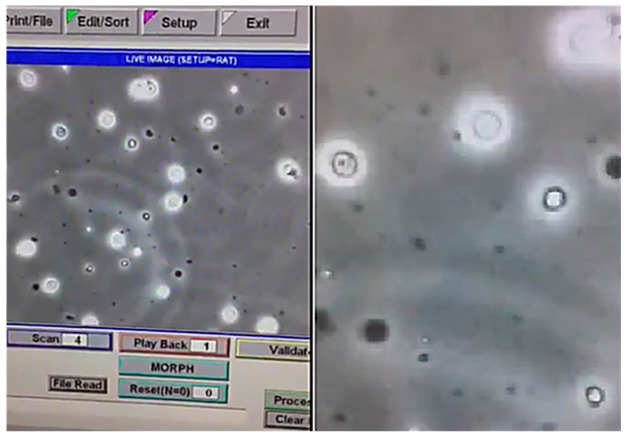
Representative live frames extracted from CASA software showing sperm cells from a cadmium-exposed rat (5 mg/kg bw). The images demonstrate a marked reduction in motile sperm, with most appearing immobile or showing abnormal morphology. The lack of visible progressive tracks and weak signal intensity suggest a significant deterioration in sperm motility and activity, consistent with cadmium-induced reproductive toxicity.

**Figure A4:**
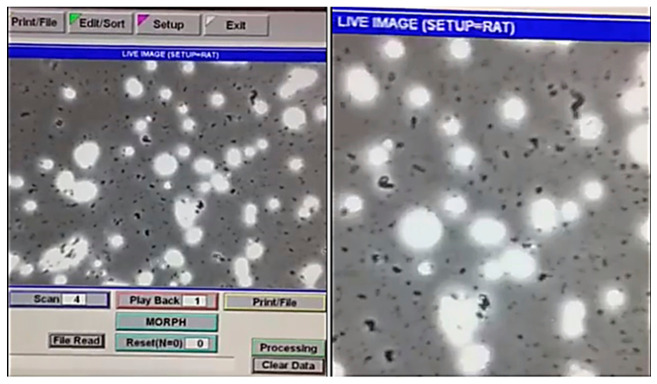
Live CASA screenshots showing sperm cells from a rat exposed to cadmium (5 mg/kg bw) and co-treated with silymarin (30 mg/kg bw). The images reveal a high number of non-motile or static spermatozoa (bright, non-tracking spots) and cellular debris, suggesting persistent structural and functional damage despite antioxidant treatment. While silymarin partially improved sperm parameters compared to cadmium-only exposure, these visual findings reflect an incomplete recovery of sperm motility and morphology under the current dose and treatment duration.

## Data Availability

The original contributions presented in this study are included in the article/Supplementary Material. Further inquiries can be directed to the corresponding authors.

## Supplementary Materials

The following supporting information can be downloaded at: https://www.mdpi.com/article/10.3390/jox16030103/s1, Figure S1: Histological sections of rat testes (H&E staining); Figure S2: Histological observations of rat kidney (H&E staining); Figure S3: Histological observations of rat liver sections (H&E staining).
