# Supplementary material for: Subchronic Cadmium-Induced Xenobiotic Toxicity in Male Wistar Rats: Antioxidant and Reproductive Protection by Standardized Silymarin with Molecular Docking Insights
Source: J Xenobiot. 2026 Jun 3;16(3):103. doi: 10.3390/jox16030103 (PMC13301452; doi:10.3390/jox16030103)
Supplement: Supplementary file 1 [file jox-16-00103-s001.zip › jox-4279479-supplementary.pdf]

Journal of
Xenobiotics

MDPI

Supplementary Materials: Subchronic Cadmium-Induced Xenobiotic Toxicity in Male Wistar Rats: Antioxidant and Reproductive Protection by Standardized Silymarin with Molecular Docking Insights

Imen Hammami, Fatma Arrari, Rahma Mahjoub, Ridha Ben Ali1, Haifa El Hentati, Afef Nahdi, Eduardo Al-berto López-Maldonado, Emna Talbi

Figure S1. Histological sections of rat testes (H&E staining)

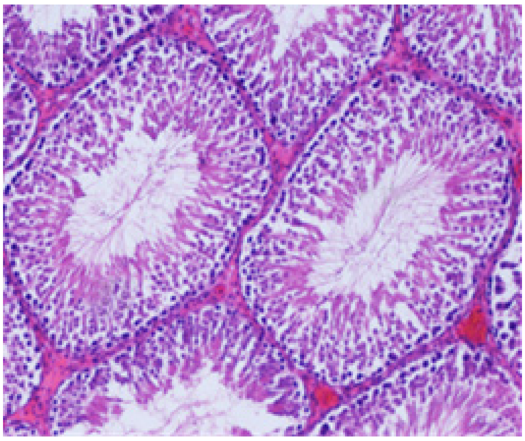

Figure S1.1. Control rat (x10)

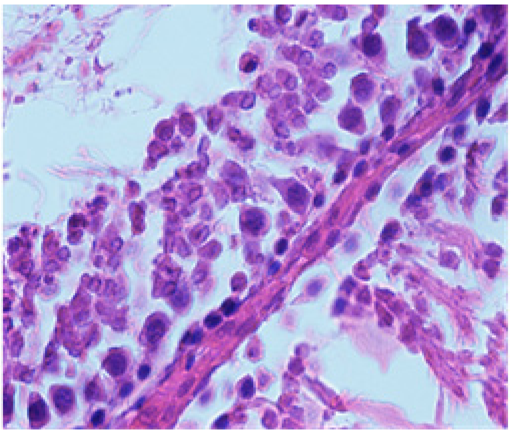

Figure S1.2. Control rat (x40)

 https://doi.org/10.3390/jox16030103

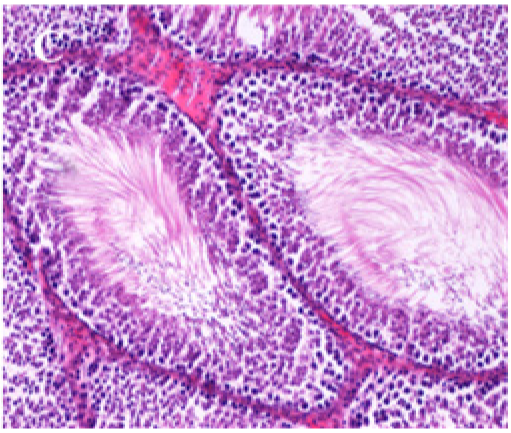

Figure S1.3. SILY rat (x10)

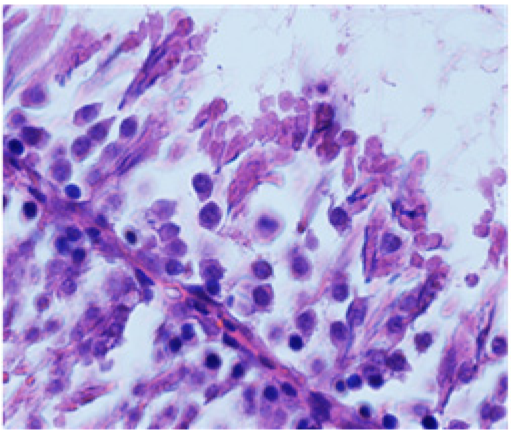

Figure S1.4. SILY rat (x40)

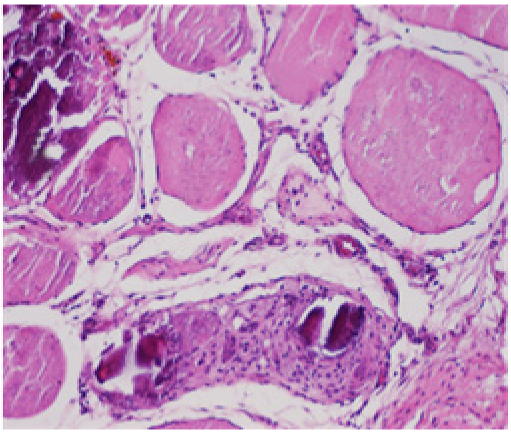

Figure S1.5. Cd rat (x10)

https://doi.org/10.3390/jox16030103

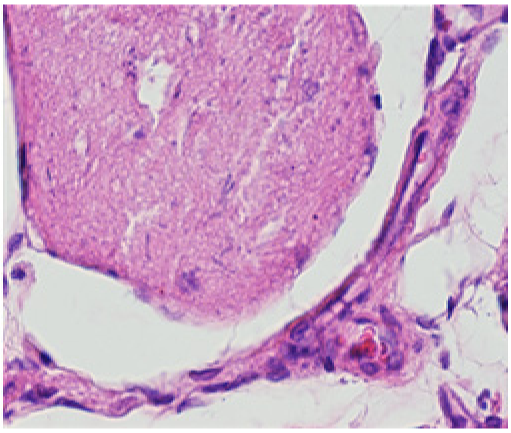

Figure S1.6. Cd (x40)

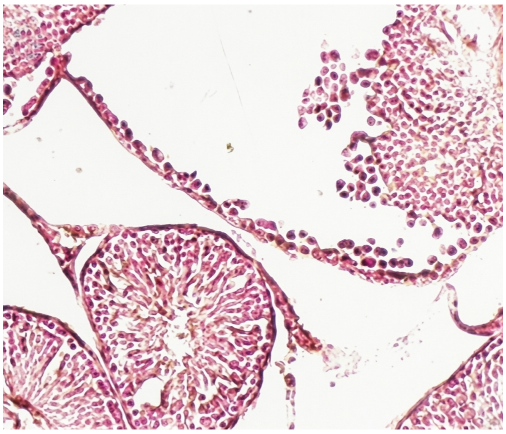

Figure S1.7. Cd+SILY (x10)

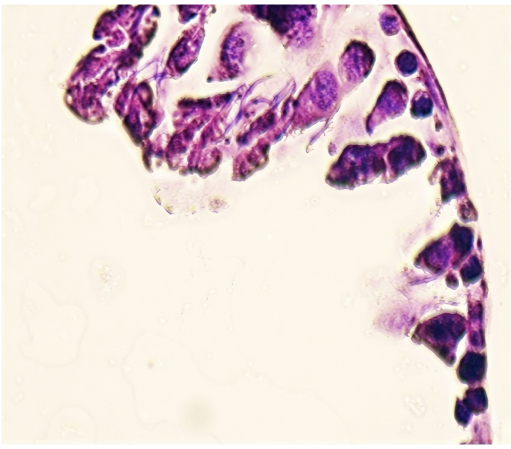

Figure S1.8. Cd+SILY (x40)

Figure S2: Histological observations of rat kidney (H&E staining).

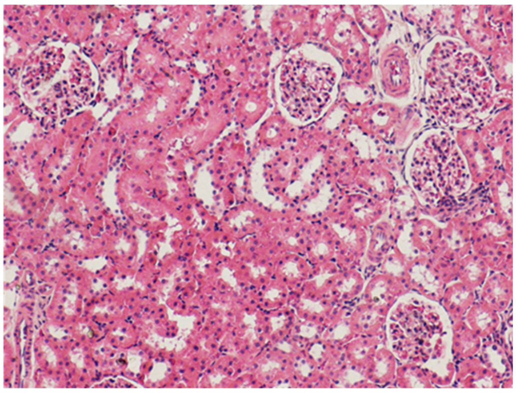

Figure S2.1. Control rat (x10)

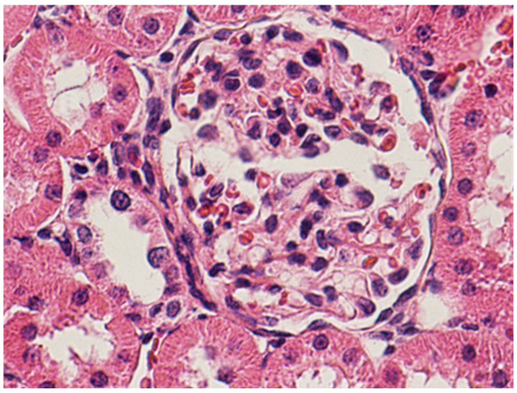

Figure S2.2. Control rat (x40)

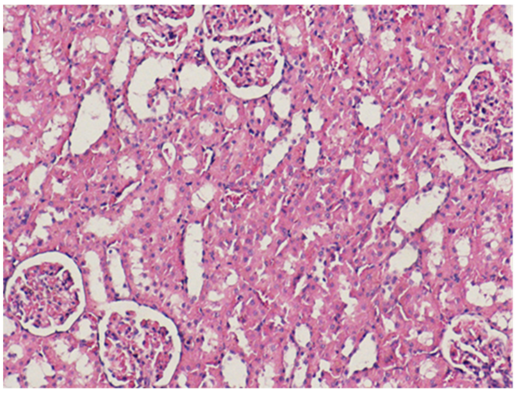

Figure S2.3. SILY rat (x10)

https://doi.org/10.3390/jox16030103

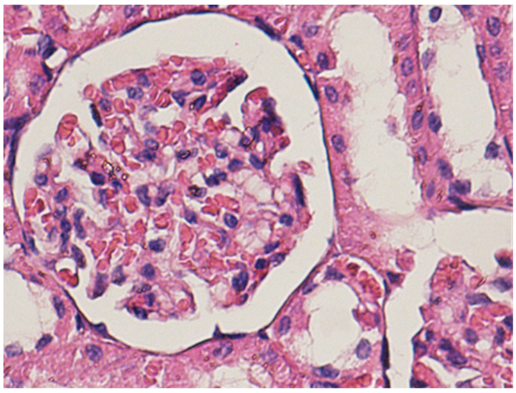

Figure S2.4. SILY rat (x40)

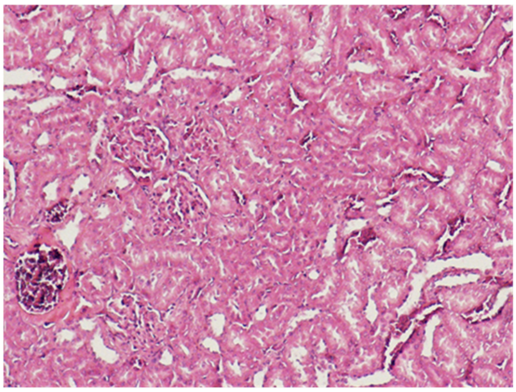

Figure S2.5. Cd rat (x10)

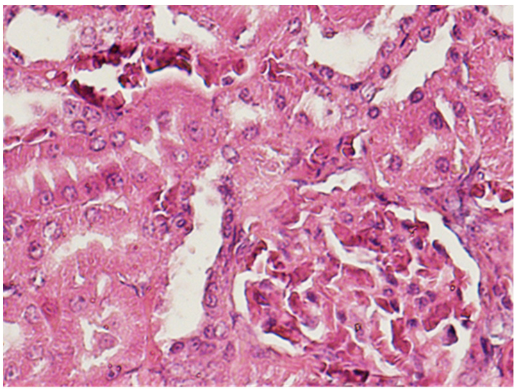

Figure S2.6. Cd (x40)

https://doi.org/10.3390/jox16030103

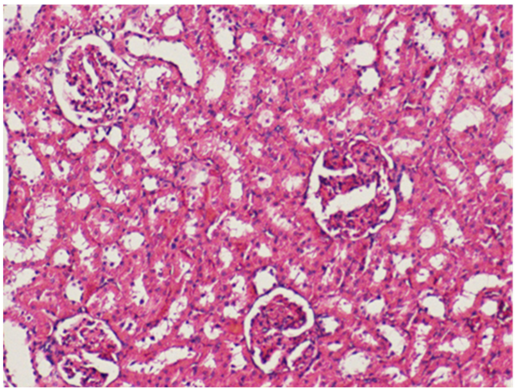

Figure S2.7. Cd+SILY (x10)

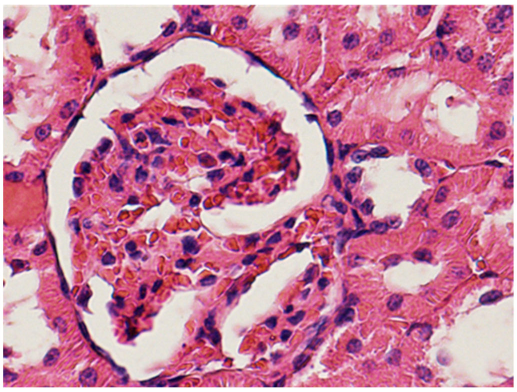

Figure S2.8. Cd+SILY (x40)

Figure S3: **Histological observations of rat liver sections (H&E staining)**.

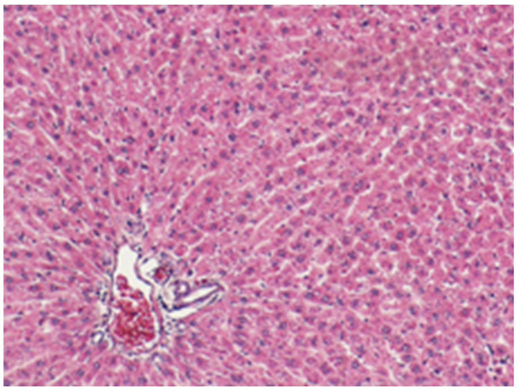

Figure S3.1. Control rat (x10)

https://doi.org/10.3390/jox16030103

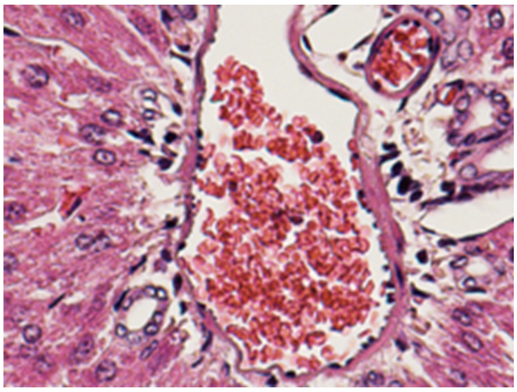

Figure S3.2. Control rat (x40)

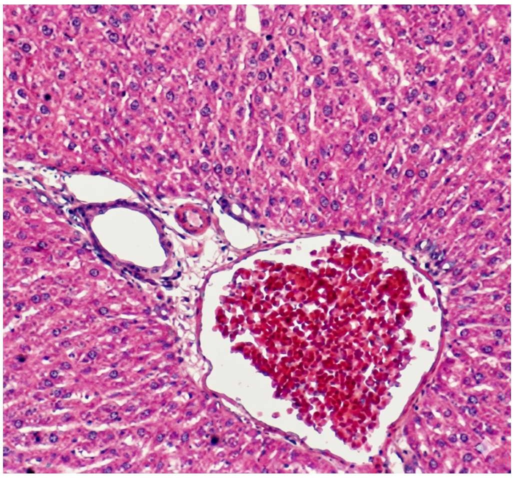

Figure S3.3. SILY rat (x10)

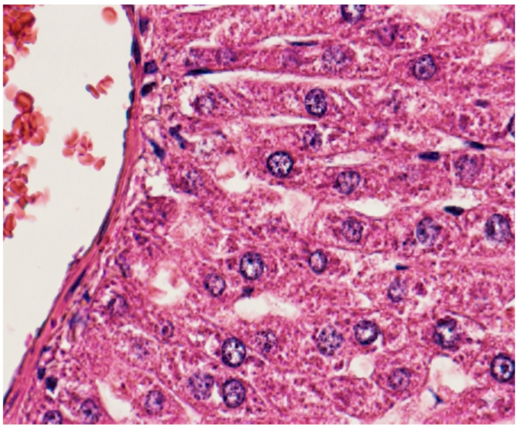

Figure S3.4. SILY rat (x40)

https://doi.org/10.3390/jox16030103

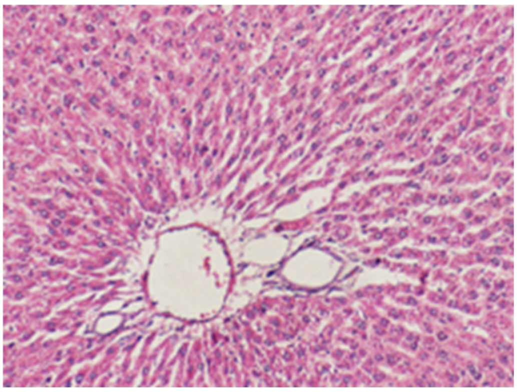

Figure S3.5. Cd rat (x10)

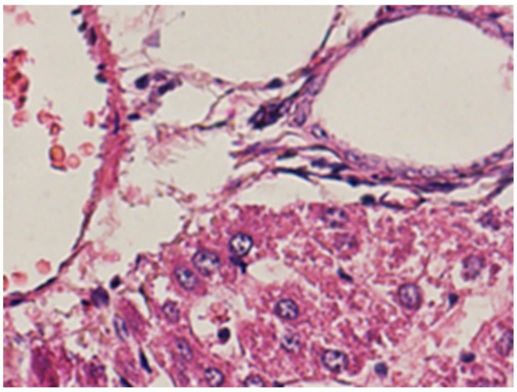

Figure S3.6. Cd rat (x40)

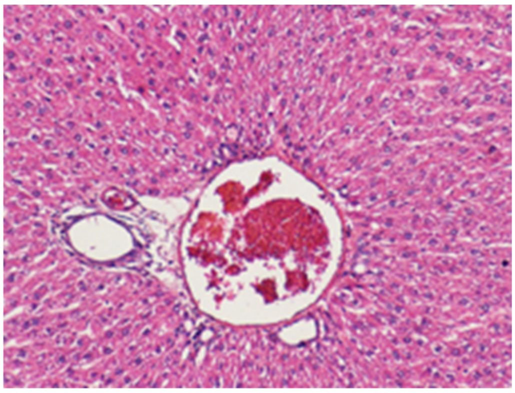

Figure S3.7. Cd+SILY (x10)

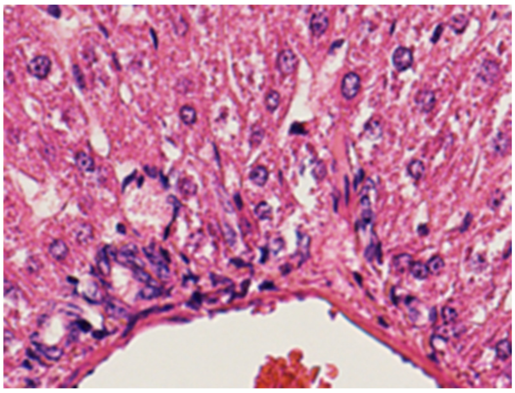

Figure S3.8. Cd+SILY (x40)

Disclaimer/Publisher's Note: The statements, opinions and data contained in all publications are solely those of the individual author(s) and contributor(s) and not of MDPI and/or the editor(s). MDPI and/or the editor(s) disclaim responsibility for any injury to people or property resulting from any ideas, methods, instructions or products referred to in the content.
